# Changes in Vertical Stratification of Neotropical Nymphalid Butterflies at Forest Edges Are Not Directly Caused by Light and Temperature Conditions

**DOI:** 10.3390/insects16010064

**Published:** 2025-01-11

**Authors:** Brian K. Oye, Ryan I. Hill

**Affiliations:** Department of Biological Sciences, University of the Pacific, 3601 Pacific Avenue, Stockton, CA 95211, USA; boye@vols.utk.edu

**Keywords:** structural causal model, community, fruit-feeding nymphalid, mediation model, Nymphalidae

## Abstract

Habitat fragmentation and land use changes threaten neotropical habitats and alter patterns of diversity at forest edges. Like other arthropod assemblages, neotropical fruit-feeding butterfly communities show strong vertical stratification within forests, with some recent work showing its potential role in speciation. At forest edges, species considered to be forest canopy specialists have been observed descending to the forest understory, with the similarity in light conditions between the canopy and understory strata at edges hypothesized to be responsible for this phenomenon. We conducted a study using standardized sampling to document and quantify this edge effect, characterize edge and forest strata, and estimate the relative contributions of temperature and light conditions to changes in nymphalid butterfly stratification at forest edges. We found strong evidence of an edge effect in these butterflies and confirmed strong differences in light and temperature, showing that the edge understory differs little from forest canopy conditions. Of 41 species common to both forests and edges, 28 shifted to have a lower canopy probability at the edge, and our model detected a decrease in canopy probability of 0.165. Furthermore, our analysis indicated the relative abundance of canopy taxa increased at the edge, and the tribes Haeterini and Morphini were especially sensitive to edge effects. However, the analyses here did not clearly implicate temperature or light magnitude in causing changes in neotropical nymphalid vertical stratification at forest edges. Instead, our results point to other mediator variables as being important for changes at tropical forest edges. From our data, edge-responsive species can be separated into two different categories, which likely relates to their resilience to anthropogenic disturbance. We also note that structural causal models have a potential place in future work on tropical conservation, given they can provide causal estimates with observational data.

## Simple Summary

Tropical forest species show strong vertical community structure, with some species preferring the canopy and others preferring the understory. Nymphalid butterflies have been used to explore this pattern with baited traps in different strata. Canopy butterflies have been observed to descend to the understory at forest edges, and this change was hypothesized to be associated with light. We used traps in the canopy and understory in Costa Rica to quantify changes in canopy preference at the edge and test if they were associated with light or temperature. Our data show the edge has a strong effect on canopy preference and there is a change in the community structure at the edge, but our data did not indicate light or temperature as direct causal effects. We identify two responses in butterflies because of the edge: some species are absent and therefore more sensitive to forest disturbances; and some species change their preference and are likely more resilient. Our analytical approach may be useful for attributing causation in observational studies.

## 1. Introduction

Habitat fragmentation and land use changes threaten neotropical habitats. The expansion of land used for cattle ranching [1] or crops such as pineapples [2,3], sugarcane [4], and soybeans [5] and various forms of resource extraction [6] have reduced or modified tropical forests in particular [7]. In addition to the potential impacts of reduced habitat area, these changes also result in habitat fragmentation, which is known to generally reduce biodiversity [8]. One way habitat fragmentation may have detrimental effects on communities is by increasing the amount of forest habitat close to forest edges.

Whether natural or anthropogenic, edges are known to impact species distributions and assemblage compositions for a wide range of taxa. Neotropical dung beetle, butterfly, amphibian, and reptile abundance and species richness are known to differ at forest edges relative to the forest interior [9,10,11]. Additionally, some taxa vary in their responses to natural edges compared to anthropogenic edges. For example, riparian edges were shown to have a greater howler monkey density, while anthropogenic edges were shown to have a greater capuchin monkey density in Costa Rica [12].

Edges often show differences in microclimate conditions relative to the adjacent habitat. These changes are called abiotic edge effects by Murcia [13]. In addition, there may also be biotic changes at edges, with abiotic and biotic explanations, respectively, referred to as direct biological edge effects and indirect biological edge effects by Murcia [13]. As described by [14], the physiological stresses from higher temperatures and lower humidity might stress animals, leading to a decrease in abundance for a given taxon. However, changes at habitat edges may also occur if, for example, the edge habitat is associated with an animal’s predators [15]. Quantifying these mechanisms is hindered by the strong associations between potentially causal biotic or abiotic explanations associated with edge habitats, such as daily temperatures and host plant presence.

Tropical butterfly communities have provided some insight into the roles of biotic and abiotic variables on community structure. Like other arthropod assemblages, a key part of tropical butterfly community structure is strong vertical stratification, in which species generally reside in either the understory or the canopy, but rarely both strata [16,17,18,19,20,21,22]. Recent work has shown that vertical stratification may serve as a barrier to gene flow [23], indicating an important potential role for vertical stratification in speciation. The importance of vertical stratification has been shown in research documenting how species richness and abundance differ in the canopy and understory by region, season, and location within a forest [17,20,24,25]. For example, DeVries et al. [26] documented increased species richness in a Costa Rica canopy compared with that in Ecuador. DeVries and Walla [18] observed canopy abundance to be higher in the early rainy season and understory abundance to be higher in other times of the year in Ecuador. DeVries et al. [27] and Walla et al. [20] showed differences in species composition between canopy and understory by habitat type. However, these studies do not address questions framed at the species level: can butterfly stratification be explained by species responses to abiotic factors?

DeVries [16] proposed that light conditions maintain the strong differences between canopy and understory groups in tropical butterfly assemblages. DeVries [16] used observational evidence that high-flying species could be captured at ground level at forest edges, where light conditions were approximately the same as in the canopy, to explain stratification. In this model, strong differences in light between the canopy and understory in a closed canopy forest would serve as a barrier to butterfly movement between the strata. Though temperature and humidity are related to light and also vary between the canopy and understory, DeVries [16] posited that light is more important for stratification. Light, temperature, and humidity would be examples of direct biological edge effects on butterflies.

Tropical butterfly stratification presents an opportunity to test whether the direct or indirect biological effects described in Murcia [13] apply to an insect assemblage and to test DeVries’ model of light driving stratification. Fruit-feeding nymphalid butterflies are particularly well suited for investigating vertical stratification because they can readily be sampled with baited traps [28]. In this study, we use baited understory and canopy traps and associated measurements of light and temperature to answer the following: (1) Is there a decrease in the canopy probability in edge habitats compared with forest habitats? and (2) To what extent are any changes in the canopy probability between the forest and edge explained by light or temperature? We predict that canopy probabilities of species decrease at edge habitats compared with forest habitats, and consistent with the model of DeVries [16], we predict that light will have a stronger association with any changes in the canopy probability than temperature.

## 2. Materials and Methods

### 2.1. Study Site and Butterfly Sampling

Butterflies were trapped at La Selva Biological Station, Heredia Province, Costa Rica from 26 May 2015 to 10 August 2016. Traps were handmade and adapted from the #2 design in Austin and Riley [29] using olive green nylon mosquito netting (1 mm × 1 mm mesh size). Traps were narrower at the bottom, and a galvanized steel (14 gauge) wire hoop with diameter of 22.86 cm was sewn into the bottom of the trap to hang the green corrugated plastic base (30.5 cm × 30.5 cm) using short pieces of polyethylene line. The top of the trap had a 27.94 cm hoop sewn in 30 cm from the top, and the loose top material was tied off with polyethylene line. The finished trap was approximately 1 m tall. Bait was held in a 350 mL plastic cup resting in a hole in the base so that 3 cm of the cup was above the base.

A total of 32 trap sites were used: 12 ridge sites, 10 valley sites, and 10 edge sites. This design was adopted to give a broad characterization of the forest habitats in terms of diversity and abiotic variation. Ridge and valley sites were combined to represent “forest” in our analyses as they are located in areas of La Selva documented as primary forest, with ridge sites located at high topographic points, and valley sites located at low topographic points with small streams. Edge sites were located where vegetation transitioned from tall forest to open low vegetation (less than 1.5 m) at natural edges such as the borders of rivers or man-made edges such as pasture, regenerating pasture, or other clearings. Each trapping location had a pair of traps, with one trap placed in the understory (mean height = 0.9 m, sd = 0.2 m) and one trap placed in the canopy (mean height = 22.7 m, sd = 5.3 m). To set canopy traps, weights attached to a polyethylene line were launched over tree branches with a Big Shot SHERRILLtree^®^ tree-climbing slingshot, after which a trap was attached and tested to avoid interference from the surrounding vegetation and allow it to be raised and lowered with low friction.

Traps were baited with rotten banana (three days old) or rotten shrimp (14 days old) to sample the nymphalid assemblage more completely than a single bait type alone [30,31,32]. Ripe banana and large frozen shrimp were obtained from local markets. Effort was made to use the same sized bananas (not small “oritos”) and same brand of shrimp during the study. From 26 May 2015 to 12 August 2015, traps were checked four consecutive days a week; from 12 September 2015 to 10 August 2016, traps were checked five consecutive days in the second week of each month. Bait was added to the traps on the first day of each sampling week and supplemented as needed to maintain attractivity.

All butterflies were photographed, marked with a unique number using a Sharpie^®^ marker, and released. Though we are focused here on nymphalids, relatively few non-nymphalids such as lycaenids, riodinids, and hesperiids were captured, but not in sufficient abundance for analysis. Species were identified using DeVries [33], Glassberg [34], and Warren et al. [35] as references. Nomenclature follows Lamas et al. [36]. Recapture rate was very low, and recaptures were excluded from analysis.

### 2.2. Temperature and Light Measurements

Temperature and light data were gathered using HOBO^®^ pendant loggers attached to the base of each trap; measurements were taken every hour or every other hour depending on storage capacity and battery life. Temperatures were recorded in Celsius and light measurements were recorded in lux. Safety lines were used to fix loggers to the traps. When traps fell due to storms and falling trees or branches, logger measurements for those times were considered missing data. The logger data for light were log transformed to facilitate the use of normal distributions and match them to insects’ light responses. Specifically, light undergoes filtering by vegetation according to Beer’s law, cumulative multiplicative effects result in lognormal random variables (see Gelman et al. [37]), and insects, like the Japanese Beetle (*Popillia japonica*), often respond to multiplicative changes in light rather than a fixed amount according to the Weber–Fechner law, likely due to sensory adaptation of their photoreceptors [38,39,40].

### 2.3. Statistical Analyses

Statistical analyses were primarily done in R [41], and all plots were made using ggplot2 [42]. Specific R packages and details are included in the text below.

#### 2.3.1. *G*-Test of Decreased Canopy Probability at Edge

To answer whether there is a decrease in canopy probability in edge habitats compared with forest habitats (question 1), we calculated *G*-test statistics and *p*-values in Excel. We performed the *G*-tests using species found at both forest and edge locations (41 species). We used canopy and understory counts to calculate canopy probability (canopy counts/total counts) and an index of change we termed “delta edge” in the *G*-test. Delta edge was calculated for each species by subtracting the canopy probability at the edge from that of the forest and describes a species change in canopy probability between habitats with positive values indicating decreased canopy probability. If there is no general pattern toward decreased canopy probability at the edge (i.e., positive delta edge), we expect half of the 41 species common to forest and edge to have positive delta edge and half to have negative delta edge. Delta edge values of 0, indicating no change, were coded as negative in the *G*-test, the sums of species with positive or negative values were used in calculating *G*, and a standard alpha of 0.05 was used.

To assess the sensitivity of canopy probability shift toward understory, we also performed *G*-tests with (i.) the genera *Caligo*, *Eryphanis*, and *Taygetis* removed since these are understory species ([23] and this study), and (ii.) additional species removed that did not have at least three individuals observed at the edge and at least three in the forest.

#### 2.3.2. Tests of Light and Temperature Among Habitats

We tested for differences in the ranked distributions for light and temperature to assess the potential for variation by habitat and stratum to explain changes in butterfly canopy probability. We plotted hourly means and 95% confidence intervals for light and temperature and used the npmv package [43] to test for microhabitat differences between strata and edge and forest habitats. We used median values across the entire study at 800, 1000, 1200, and 1400 h for the four habitat–stratum combinations. We used the ssnonpartest function with the Wilks’ lambda-type test statistic for the analysis [44].

#### 2.3.3. Bayesian Model—Definition of Causal Relationships

**Figure 1:**
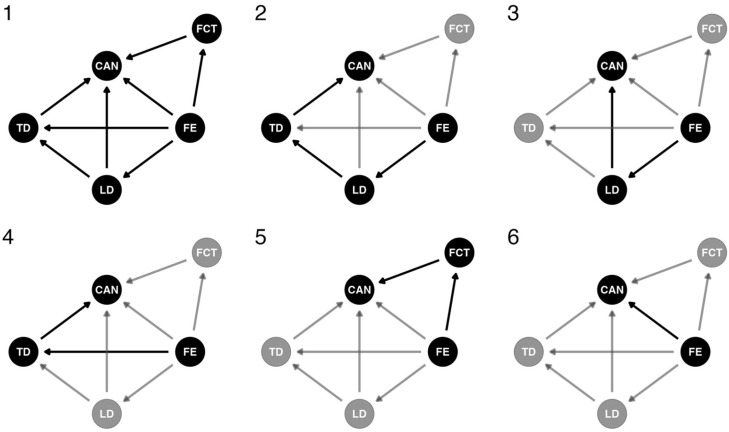
Structural causal model. Abbreviations are *FE* for forest edge, *LD* for log light difference, *TD* for temperature difference, *FCT* for forest canopy tendency, and *CAN* for canopy observations. Bold indicates specific pathways as follows: Panel (**1**) shows the complete graph, panel (**2**) shows the effect of *FE* on *CAN* through *LD* and *TD*, panel (**3**) shows the effect of *FE* on *CAN* through *LD*, panel (**4**) shows the effect of *FE* on *CAN* through *TD*, panel (**5**) shows the effect of *FE* on *CAN* through *FCT*, and panel (**6**) shows the effect of *FE* on *CAN* without mediator variables.

We used an individual observation approach applying a Bayesian mediation model to provide answers to both questions 1 and 2. A mediation model has a predictor variable (i.e., “exposure variables” in epidemiology literature [45]), an outcome variable, and one or more mediator variables. We created a structural causal model (a type of directed acyclic graph [46]) to represent causal relationships proposed by DeVries [16] (Figure 1). Structural causal models are diagrams used to represent causal relationships relating predictor and outcome variables regardless of functional form [47], and they can aid in mediation model specification by showing how the effects of a given variable may be decomposed into direct and indirect effects [48]. Causal relationships between variables, represented as nodes, are indicated with directed edges (arrows in Figure 1). We estimated various edge effect contributions to butterfly canopy probability using a potential outcomes framework with the mediation model [49].

In our causal representation, the forest edge indicator *FE* is a binary predictor variable, with *FE_i_* = 1 for edge locations and *FE_i_* = 0 for forest locations, for every individual observation *_i_*. Both ridge and valley locations were defined as forest locations in the mediation model. Whether a location is in primary forest or at a forest edge affects *LD_i_*, the log light difference between the strata, *TD_i_*, the temperature difference between the strata, and *FCT_i_*, the forest canopy tendency for a given observation (species composition may change at forest edges) [9]. In the mediation model, the variable *FCT* represents baseline forest canopy tendency for a given butterfly observation based on species. To visualize the potential issue of composition confounding our effect estimates [16], we made a graphic of tribes as a visual representation of habitat stratum patterns present in the data. This graphic plots relative abundance by tribe.

The light conditions also should affect the temperature conditions at a given location as represented in Figure 1 by the edge going from *LD* to *TD*. Finally, the forest edge *FE* may impact canopy observations *CAN*, with *CAN_i_* = 1 for an individual captured in a canopy trap, and *CAN_i_* = 0 for an individual captured in an understory trap. *FE* may affect *CAN* indirectly through nodes *LD*, *TD*, or *FCT*, or directly with no mediator variable(s). For example, panels 2–5 in Figure 1 indicate types of indirect effects. Panel 6 in Figure 1 shows the direct effect of the forest edge on canopy probability. The sum of all causal paths from *FE* to *CAN* is the total causal effect (*TCE*) of *FE* on *CAN.*

It is important to note that in structural causal models, mediator variables can act as both predictor and outcome variables, depending on their relationships with connected variables. For example, *LD*, *TD*, and *FCT* in Figure 1 act as both predictor and outcome variables since they are intermediate nodes on causal paths from *FE* to *CAN*. In contrast, *FE* is only a predictor, and *CAN* is only an outcome.

#### 2.3.4. Bayesian Model—Causal Effect Definitions

We calculated causal effects of *FE* on *CAN* using the potential outcomes framework (see Daniel et al. [50] and Imai et al. [49]). For example, *LD*(*FE* = 1), abbreviated *LD*(1), indicates the potential outcome for the log light difference expected for a trap at the forest edge; *TD*(*FE =* 1, *LD*(*FE =* 0)), abbreviated *TD*(1, *LD*(0)), indicates the temperature difference expected for an edge trap with forest light conditions; and *FCT*(*FE* = 0), abbreviated *FCT*(0), indicates the forest canopy tendency estimated for forest conditions.

Light effect was defined as the change in canopy probability resulting from differences in log light conditions between the edge and forest habitats. DeVries’ view that decreasing light differences between the canopy and understory at the edge explain canopy probability changes would be a negative light effect on canopy probability. The light variable was modeled based on the edge indicator variable *FE*; values for *TD* were held at values expected for forest locations to adjust for effects of temperature. The effect was defined as follows:

(1) Light Effect=E{CAN(1,LD(1), TD(0, LD(0)), FCT(0))}−E{CAN(1,(0), TD(0, LD(0)), FCT(0))}.

The temperature effect was defined as the sum of all effects resulting from changes to *TD*.

(2) $$Temperature\;Effect=E\left\{CAN\left(1,LD\left(1\right),\;TD\left(1,\;LD\left(1\right)\right),\;FCT\left(0\right)\right)\right\}-E\left\{Y\left(1,LD\left(1\right),\;TD\left(0,\;LD\left(0\right)\right),FCT\left(0\right)\right)\right\}$$

We defined species composition effect (*SCE*) as the change in canopy probability resulting from differences in *FCT* between the forest and edge habitats.

(3) Species Composition Effect=E{CAN(1,LD(1), TD(1, LD(1)), FCT(1))}−E{CAN(1,LD(1), TD(1, LD(1)), FCT(0))}

We defined edge effect as *TCE* − *SCE*, which serves as an estimate of the edge effect described in DeVries [16]. This effect holds species tendencies constant between edge and forest locations, so changes in species composition do not contribute to its value. Equations for other causal estimates are provided in Equations (S29)–(S37).

#### 2.3.5. Bayesian Model—Mediation Model Specification

Linear regression models with normally distributed errors were used for *LD*, *TD*, and *FCT*, and a logistic regression model was used for *CAN* (see Equations (S25)–(S28)). We used the rriskDistributions R package [51] to fit normal distributions to the logit-transformed posterior probability quantiles based on posterior distributions of canopy probability from a previous study [23]. Flat priors were specified for the regression coefficients and standard deviations. We used the bayesplot [52] and caret [53] packages to assess model fit for the mediation model regressions.

#### 2.3.6. Bayesian Model—Missing Data

The abiotic data (temperature and light measurements) had missingness from logger displacement due to storms and a relative lack of loggers, especially in the first half of the study (Figure S1). For example, we had insufficient HOBO^®^ loggers to record temperature and light measurements for trap locations 10, 18, 22, 29, and 30 (one forest edge location, one valley location, and three ridge locations, respectively). Consequently, we could not match some butterfly captures to temperature or light measurements. The *FCT* variable also had missingness; canopy preferences for some taxa, like *Adelpha*, were not reported by Nice et al. [23].

The most traditional approach to missing data is listwise deletion/complete case analysis [54]. If the probability of missingness is equal for all observations (missing completely at random, MCAR), parameters estimated using listwise deletion will have low bias [55]. However, when the MCAR assumption is less reasonable, listwise deletion loses this appealing property and still requires that one dispose of information. Unbalanced missingness by time and trap location for our autocorrelated temperature and light data series made the MCAR assumption unreasonable.

We specified a joint mediation model/missing data model to make better use of our data. Incorporating time-related structures for our abiotic data and a taxonomic measure for the canopy preference covariate made our missing at random assumption more reasonable. Models were specified in a Bayesian framework to permit use of prior information [56]. In this context, missing data are parameters conditional on observed data estimated simultaneously with the mediation analysis model parameters as part of a joint posterior probability distribution [57,58].

The functional form of the missing data model had two modules as follows. First, the missing abiotic data were modeled with a state space representation [59] to leverage the time series nature of the data (Equations (S1)–(S21)) [55]. The state of the system was represented as a 12-dimensional vector, with one element for each habitat/stratum type series (e.g., edge understory log light and edge canopy temperature). This state was related to the logger values according to their type. We used broad, informative priors for mean temperatures and mean log light to constrain the range of possibilities based on previously recorded temperature values from La Selva [60] and control values for log light from Dominioni et al. [61]. Second, missing *FCT* values for species were modeled hierarchically by tribe (Equations (S22)–(S24)). While conceptually distinct, the mediation model and the missing data model informed a joint posterior distribution, allowing information to flow freely between the modules.

#### 2.3.7. Bayesian Model—Model Fitting

Model fitting was performed using CmdStan (version 2.34.0) [62,63]; we used Stan’s No-U-Turn sampler for the analysis model and fitted the missing data assessment models using Stan’s optimization algorithm. Posterior convergence was satisfactory based on CmdStan’s diagnose utility for R-hat and effective sample sizes. Further details and Stan code for the models are provided in the Supplementary Materials file.

## 3. Results

### 3.1. Trapping Results, Light, and Temperature

#### 3.1.1. Butterfly Observations

**Figure 2:**
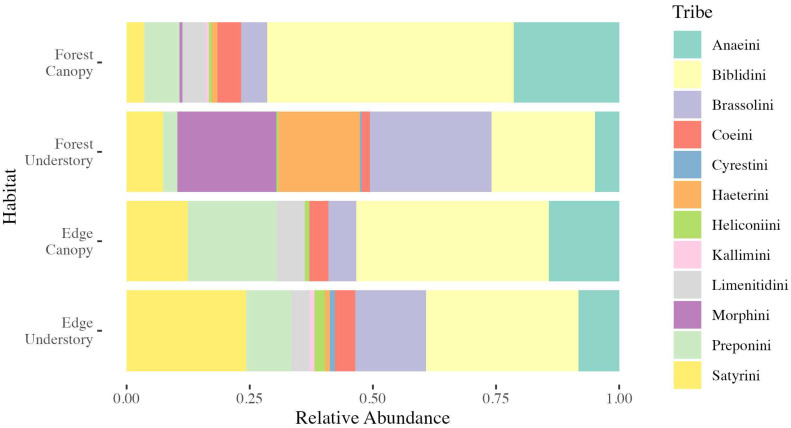
Tribe relative abundances by habitat and strata type. Changes in tribe abundance in different strata and habitat shown here are related to the species composition effect in the mediation model. X-axis is provided as a rough guide. For specific relative abundances, see Supplementary Table S1.

In total, we trapped 710 unique individuals distributed among 92 species across 12 tribes. There were 14 recaptures that were excluded from the analysis. Of these individuals, 299 were at edge trap sites and 411 were at forest trap sites (213 in valleys and 198 in ridges). Although forest and edge locations were similar in having high relative abundances for Biblidini, Morphini were captured exclusively at forest traps, and Haeterini showed a similar capture pattern, with only two individuals recorded for edge traps (Figure 2). Additionally, Preponini and Satyrini both had a higher relative abundance in edge locations relative to the forest locations (Figure 2).

#### 3.1.2. Temperature and Light Differences

**Figure 3:**
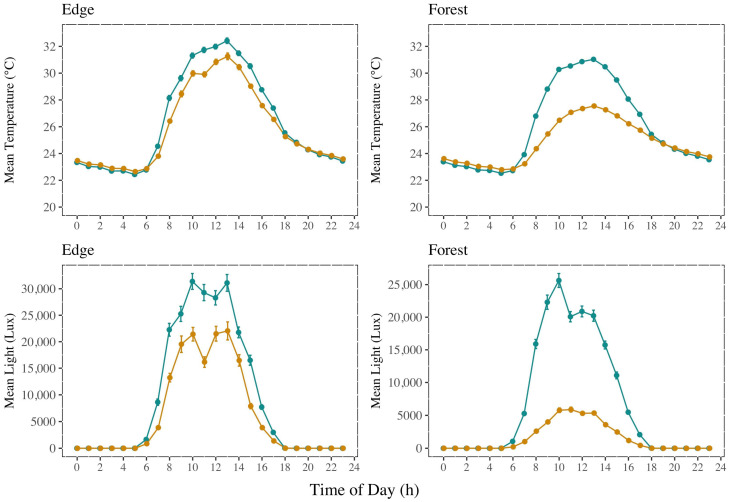
Mean temperature and light by hour; bars indicate 95% confidence intervals. Canopy values are in cyan, and understory values are in orange.

The logger data showed clear differences between the understory and canopy, as well as between habitat types (Figure 3). As expected, canopy trap loggers registered brighter and hotter conditions during the day, but canopy trap loggers were cooler than understory loggers from approximately 8pm to 6am. Peak temperatures were roughly similar among the different habitats, though edge canopy traps tended to be slightly hotter than canopy traps for forest locations.

The nonparametric multivariate test of abiotic conditions among edge and forest habitats confirmed significant differences between the forest understory and the other three groups (forest canopy, edge canopy, and edge understory; *p* < 0.05, see Table 1). The edge understory and edge canopy did not differ in abiotic conditions, and the edge understory was similar to the forest canopy conditions (Table 1), consistent with the patterns of temperature and light seen in Figure 3.

### 3.2. Species Canopy Probability Changes

#### 3.2.1. Changes in Species Canopy Probability at Forest Edges (*G*-Tests)

Of the 41 species we observed at both edge and forest locations (Table 2), 28 showed decreased canopy probability at edge locations, significantly more than the 20.5 expected (*G*-test of independence, *G* = 5.6, df = 1, *p* = 0.018). Removing the genera *Caligo*, *Eryphanis*, and *Taygetis*, which are genera known only from the understory in this study and other studies [23], reduced the number of species common to the edge and forest locations to 37, with 28 having decreased canopy probability at edge locations (*G* = 10.2, *p* = 0.0014). Removing species that have fewer than three observations in both edge and forest habitats with *Caligo* and *Taygetis* removed resulted in 14 species common to edge and forest locations, with 12 observed with decreased canopy probability at the edge locations (*G* = 7.9, *p* = 0.0049).

#### 3.2.2. Forest Edge Causal Effects on Canopy Probability (Mediation Model)

**Figure 4:**
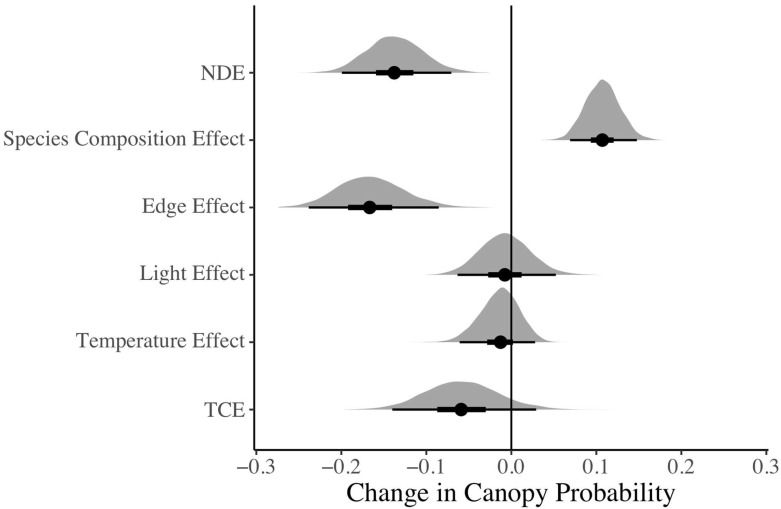
Estimated effects from the mediation model. Narrow outer lines indicate 95% credible intervals; thick inner lines indicate 50% credible intervals; and points indicate posterior means. NDE = natural direct effect (effect of edge on canopy probability while holding light and temperature differences and forest canopy tendency constant) and TCE = total causal effect (effect of edge on canopy probability along all paths).

We detected a strong edge effect of −0.165 on the canopy probability (with a 95% credible interval of [−0.239, −0.085]) as a result of the forest edge after adjusting for differences in species canopy tendencies (Figure 4). The natural direct effect (NDE) of the forest edge was −0.137 (with a 95% credible interval of [−0.199, −0.071]) and is the effect of the edge on canopy probability while holding light and temperature differences and the forest canopy tendency constant (Figure 1, panel 6). The species composition effect was 0.107 (with a 95% credible interval of [0.069, 0.148]), as a result of the average forest canopy tendency increasing at the edge. The light effect on canopy probability (−0.007, with a 95% credible interval of [−0.063, 0.052]) and the temperature effect on canopy probability (−0.014, with a 95% credible interval of [−0.061, 0.028]) were slightly negative but close to and overlapping with zero. The total causal effect (TCE) was −0.058 (with a 95% credible interval of [−0.140, 0.029]) and overlapped with zero because NDE and SCE were similar in magnitude with opposite signs, while the light effect and temperature effect were close to zero (Figure 4).

Posterior predictive checks for the mediation model linear regressions and a calibration plot for the mediation model logistic regression showed our regressions generated reasonable expected values and predictive distribution dispersions (Figure S2).

Posterior predictive checks of the missing data model (Figure S3) did not show a substantial incompatibility of the effect estimates calculated from completed and replicated data [64], suggesting our missing data model was reasonable. A visual inspection of the temperature and light time series estimates aligned with the observed data (Figures S4–S14).

## 4. Discussion

This is the first study to show changes in neotropical nymphalid canopy preferences at the forest edge and associate them with standardized measurements of temperature and light. To answer our questions, we needed to document differences in butterfly canopy probability, as well as the variation in abiotic variables between the understory and canopy among forest and edge locations.

### 4.1. Edge Effect on Canopy Probabilty and Abiotic Variables

Our *G*-test and mediation model both confirm DeVries’ observations [16] of an edge effect on canopy probability. This can be seen in both the positive delta edge index for 28 of the 41 species common to both forest and edge locations, indicating a decreased canopy probability by species at the edge, and in the non-zero edge effect of −0.165, which represents a decrease in the individual canopy probability at the edge after adjusting for species composition.

We also found, when holding other factors constant, the change in species composition between forest and edge habitats increased the relative abundance of canopy taxa at the edge. This was seen in the positive species composition effect (SCE = 0.107) in the mediation model, indicating there are more captures of species with high *FCT* at the edge. This may be due to the complete absence of Morphini at edge traps and a large decrease in relative abundance of Haeterini at edge traps (Figure 2). This is not surprising as these species seem to generally prefer the forest understory and avoid edge habitats, which, in the case of Haeterini, is possibly due to their use of ground effect-based gliding flight [65]. Similarly, canopy specialists may be constrained in primary forest due to relative lack of flight paths in the forest understory [19]. Our detection of an SCE differs from the lack of species composition differences in the study of Gueratto et al. [66], who studied five-to-six-meter-wide forest edges created by unpaved trails. This difference between studies is likely due to differences in the forest edge width between the studies.

Our data also clearly show that temperature and light in the understory and canopy strata varied according to the different habitats. The edge strata were distinct from the forest understory but could not easily be distinguished from the forest canopy locations. Temperatures and light intensities were high in edge habitats for both the understory and canopy traps (Figure 3). This similarity between the edge understory and the forest canopy temperatures aligned with previous descriptions for gap and canopy locations [67]. Canopy traps for all locations tended to be slightly warmer than understory traps for both edge and forest habitats (Figure 3), but this general pattern reversed at night.

### 4.2. Edge Effect on Canopy Probability Is Not Explained by Light and Temperature Variation

Despite confirming strong differences in abiotic variables and detecting a robust edge effect on canopy probability, the mediation model results did not indicate light or temperature as drivers of butterfly canopy probability. Estimates of light and temperature effects had 50% credible intervals overlapping with zero (Figure 4), showing that decreases in light and temperature differences do not explain the decrease in canopy probability at the forest edge. Since our mediation model adjusts for species composition using *FCT*, this result is not due to differences in baseline species’ tendencies for strata. In contrast, the credibly negative natural direct effect (NDE) of the forest edge on canopy probability suggests that factors other than light and temperature magnitudes are affecting butterfly vertical stratification at the edge.

Although our study found temperature and light intensity had little effect on butterfly canopy probability, it does not fully reject a possible role for either in nymphalid vertical stratification. For example, our analyses did not assess whether stratification within forests is associated with light or temperature. With respect to forest edges, potential differences may be due to unmeasured aspects of light or the true mechanisms being different from DeVries’ hypothesis. For example, a model of butterfly stratification based on temperature or light thresholds rather than the difference between canopy and understory conditions could explain our observations. This would be expected if taxa preferring the canopy make flight decisions based on avoiding poor conditions and devote comparatively little effort to searching for optimal conditions. Our results could also be explained by butterfly decision making based on unmeasured quantities. For example, although nymphalid butterflies are believed to have roughly similar color receptors among various taxa [68], the spectral composition of light in different habitats [69] may be important to butterfly stratification by impacting foraging, predation, or host plant identification, but our data do not directly include spectral information.

Another factor to consider before ruling out light and temperature as causes for vertical stratification is the low flexibility of our mediation model. While our model fit assessments indicated our regressions were suitable for effect estimation (Figure S3), other model forms might better reflect potential relationships between light, temperature, habitat type, and butterfly canopy observations. Although our use of species-level canopy preferences accounted for some of the variation between species, our model did not account for potential variation in regression slopes among species. We expect species may differ in their susceptibility to abiotic factors and habitat structure due to differences in their flight patterns and daily activity patterns, as shown in several palatable and unpalatable tropical species [70,71,72]. Furthermore, the relationship between our abiotic variables and log odds might not be linear; a more flexible model with splines could be worth investigating in future research.

Aside from abiotic variables, the dominant change at edges involves the vegetation, which was unmeasured in this study but very likely affects canopy probability. For example, the structure of vegetation related to butterfly courtship (i.e., perching/patrolling) or larval host plant distributions may impact canopy probabilities [73]. Two common species at the study site that illustrate how larval resources and male courtship may affect understory presence at forest edges are *Catonephele orites* and *Nessaea aglaura*, both of which are from the tribe Biblidini. Both species can generally be found in forest canopies (DeVries [16] and data herein) but are well documented in visiting light gaps, where they can be observed in the understory [26,33]. The species differ in male courtship behavior, with males of *C. orites* patrolling the canopy while males of *N. aglaura* are generally found perching within the forest understory [33]. Despite the differences in courtship, the species overlap on larval host use, both using *Alchornea costaricensis* (Euphorbiaceae), and females can be observed ovipositing on plants in the understory (RIH, personal observation and [33]). This example is not likely limited to Costa Rica since both genera are widely distributed.

Our mediation model results clearly show an effect on the canopy probability at the edge that is not explained by our measured variables. Future research could build on our approach using structural causal models by partitioning our NDE into causal paths through additional mediators. Humidity, for example, was not measured here, but would be an obvious mediator variable between the *FE* and *CAN* nodes since it changes with vegetation, and butterfly assemblages are known to vary with humidity [30]. The spectral distribution of light, as mentioned above, strongly changes at forest edges depending on the angle of the sun (i.e., woodland shade versus large gaps [69]), creating different signaling environments and potentially affecting courtship or predation. Resources could also be mediators. For example, increased puddling resources at edge locations may draw individuals searching for sodium chloride or amino acids [74,75], particularly for inland locations given the lower sodium availability [76]. Vertebrate urine and dung could also be responsible [75], as vertebrate compositions at edges often differ from those of the forest interior [11,77]. These are just a few of the potential mediators relevant to butterfly natural history.

Furthermore, while our abiotic data are better matched to our butterfly data than in typical community ecology studies, they remain an imperfect measure of the conditions experienced by the butterflies, because we had no way to associate the capture of an individual to specific temperature or light values at the exact moment the individual entered the trap. For example, our logger data had an hourly resolution at best, and a canopy-preferring individual could descend to the understory when the light differences between strata are minimal (i.e., at dawn or dusk), despite large light differences for the day overall. Still, our mediation model’s use of temperature and light data is far finer in resolution than common community ecology analyses modeling assemblages as functions of summarized habitat or plot properties, for example, the study of Melo et al. [78]. Future research could focus on using finer spatial and temporal scales to more closely link abiotic variables to observations of individual butterflies. Sampling multiple times per day for more consecutive days could provide data on individual marked butterflies, something that was not possible in this study, in which samples were taken five days a month.

### 4.3. Conservation Implications

Based on our data, we think it is useful to separate edge-responsive species into two different categories, which have implications for resilience in the face of anthropogenic habitat alteration. In the first category are species absent or nearly absent from edge locations. This includes tribes like Haeterini and the *Morpho* species sampled in our study, which rely on the shaded forest understory. In the second category are species typically defined as canopy species like *N. aglaura* that can be captured in the understory, as first described by DeVries [16]. The first category of species is likely to be much more vulnerable to population declines resulting from the loss of primary forest and fragmentation, as described by Horner-Devine et al. [14] and Daily and Ehrlich [79]. Furthermore, this is likely a broadly general phenomenon across neotropical forests at least, given that canopy preferences are strongly correlated with subfamilies and tribes [16] and that the groups studied here are widespread. In contrast, the second category of species may be more resilient to changes in forest habitats and fragmentation, assuming larval food plants and other interspecific interactions are not affected [9].

It is also worth noting that our analytical approach provides benefits for tropical butterfly conservation research. Since it is difficult to perform manipulative controlled experiments with tropical butterfly systems, researchers often rely on observational data. A formal causal inference approach with observational data produces effect estimates consistent with policy interventions to the extent the SCM captures the most important causal paths for this study system [46]. Non-causal analyses of observational data lack this consistency, so while they can be used for making predictions given a stable causal system, they are less suited to assessing the consequences of conservation policy. Policy decisions are interventions on natural environments, so causal estimates of, for example, the effects pioneer tree species [9] have on fruit-feeding butterfly assemblages are required to reliably predict the consequences of large-scale pioneer tree plantings.

## 5. Conclusions

In conclusion, this is the first study to quantify changes in canopy tendency in nymphalid butterfly species and closely link individual butterfly observations to light and temperature conditions. We found a robust effect of forest edges on the canopy probability that was not explained by light and temperature differences and that was independent of changes in species composition despite clear differences in these factors between forest and edge locations. This study paves the way for further investigations of the causal mechanisms responsible for vertical stratification, and we also note that formal causal inference methods are useful for accurate predictions of conservation policy decisions based on observational data.

## Figures and Tables

**Table 1:** Results of pairwise tests (npmv, version 2.4.0 [43]). Significant differences (*p* < 0.05) in rank distributions are indicated by *. Other abbreviations are as follows: NS, not significant; FC, forest canopy; FU, forest understory; EC, edge canopy; EU, edge understory.

	FC	FU	EC	EU
FC	-			
FU	*	-		
EC	NS	*	-	
EU	NS	*	NS	-

**Table 2:** Counts, canopy probabilities, and delta edge values for the 41 species common to edge and forest locations. Species with positive delta edge values are indicated in bold and are the species that show decreased canopy probability at edge compared with forest locations. EC, edge canopy, EU, edge understory, FC, forest canopy, FU, forest understory.

Species	EC	EU	FC	FU	Edge Canopy Probability	Forest Canopy Probability	Delta Edge
*Adelpha iphiclus*	5	6	7	0	0.4545	1.0000	**0.5455**
*Adelpha naxia*	1	0	1	0	1.0000	1.0000	0.0000
*Archaeoprepona demophon*	2	14	1	3	0.1250	0.2500	**0.1250**
*Archaeoprepona demophoon*	1	0	2	0	1.0000	1.0000	0.0000
*Archaeoprepona meander*	0	2	0	1	0.0000	0.0000	0.0000
*Caligo atreus*	0	4	0	20	0.0000	0.0000	0.0000
*Caligo brasiliensis*	0	9	0	12	0.0000	0.0000	0.0000
*Catoblepia orgetorix*	0	2	1	24	0.0000	0.0400	**0.0400**
*Catonephele numilia*	4	17	4	2	0.1905	0.6667	**0.4762**
*Catonephele orites*	1	13	14	12	0.0714	0.5385	**0.4670**
*Cissia confusa*	1	1	3	2	0.5000	0.6000	**0.1000**
*Colobura annulata*	1	1	5	2	0.5000	0.7143	**0.2143**
*Dryas iulia*	0	4	1	0	0.0000	1.0000	**1.0000**
*Dulcedo polita*	0	2	1	26	0.0000	0.0370	**0.0370**
*Epiphile adrasta*	0	1	1	0	0.0000	1.0000	**1.0000**
*Eryphanis lycomedon*	0	7	0	2	0.0000	0.0000	0.0000
*Fountainea eurypyle*	0	1	1	0	0.0000	1.0000	**1.0000**
*Hamadryas amphinome*	4	0	4	0	1.0000	1.0000	0.0000
*Hamadryas arinome*	1	1	10	3	0.5000	0.7692	**0.2692**
*Hamadryas laodamia*	9	1	12	0	0.9000	1.0000	**0.1000**
*Historis odius*	2	2	2	0	0.5000	1.0000	**0.5000**
*Magneuptychia gomezi*	1	1	2	1	0.5000	0.6667	**0.1667**
*Megeuptychia antonoe*	1	0	0	1	1.0000	0.0000	−1.0000
*Memphis artacaena*	1	1	2	0	0.5000	1.0000	**0.5000**
*Memphis cleomestra*	1	0	2	1	1.0000	0.6667	−0.3333
*Memphis mora*	0	1	1	0	0.0000	1.0000	**1.0000**
*Memphis moruus*	11	3	3	0	0.7857	1.0000	**0.2143**
*Myscelia cyaniris*	3	14	0	1	0.1765	0.0000	−0.1765
*Myscelia leucocyana*	6	2	5	0	0.7500	1.0000	**0.2500**
*Nessaea aglaura*	0	5	7	29	0.0000	0.1944	**0.1944**
*Nica flavilla*	1	0	1	0	1.0000	1.0000	0.0000
*Opsiphanes cassina*	6	0	4	1	1.0000	0.8000	−0.2000
*Pareuptychia metaleuca*	0	5	1	3	0.0000	0.2500	**0.2500**
*Prepona laertes*	16	2	7	0	0.8889	1.0000	**0.1111**
*Pyrrhogyra neaerea*	0	1	5	1	0.0000	0.8333	**0.8333**
*Pyrrhogyra otolais*	1	1	2	0	0.5000	1.0000	**0.5000**
*Taygetis thamyra*	0	15	0	5	0.0000	0.0000	0.0000
*Temenis laothoe*	3	1	7	2	0.7500	0.7778	**0.0278**
*Tigridia acesta*	0	2	1	2	0.0000	0.3333	**0.3333**
*Zaretis isidora*	0	2	5	3	0.0000	0.6250	**0.6250**
*Zaretis itys*	0	3	7	7	0.0000	0.5000	**0.5000**

## Data Availability

Data are available at Dryad (https://doi.org/10.5061/dryad.v41ns1s63, accessed on 5 January 2025) and from the authors.

## Supplementary Materials

The following supporting information can be downloaded at: www.mdpi.com/10.3390/insects16010064/s1, Figure S1: Missingness map for logger locations; Figure S2: Posterior predictive checks comparing density estimates for observed data and predictive distribution samples for Forest Canopy Tendency (FCT), Log Light Difference (LD), and Temperature Difference (TD) regressions. Calibration plot for CAN (bottom) compares binned CAN observations to model expected values for CAN; Figure S3: Posterior predictive checks and posterior predictive *p*-values (1000 replicates) for missing data model assessment; Figure S4: Temperature and log illuminance state estimates for edge habitat; Figure S5: Temperature and log illuminance state estimates for ridge habitat; Figure S6: Temperature and log illuminance state estimates for valley habitat; Figure S7: Temperature and log illuminance series for traps 1–4; Figure S8: Temperature and log illuminance series for traps 5–8; Figure S9: Temperature and log illuminance series for traps 9–12; Figure S10: Temperature and log illuminance series for traps 13–16; Figure S11: Temperature and log illuminance series for traps 17–20; Figure S12: Temperature and log illuminance series for traps 21–24; Figure S13: Temperature and log illuminance series for traps 25–28; Figure S14: Temperature and log illuminance series for traps 29–32; Table S1: Butterfly capture counts for edge canopy (EC), edge understory (EU), valley canopy (VC), valley understory (VU), ridge canopy (RC), and ridge understory (RU) locations; Equations (S1)–(S28): Main model equations. Design matrix A matches trap series to their habitat-stratum state estimates; Equations (S29)–(S37): Estimated causal effects; Code S1: Main analysis model Stan code and algorithm details; Code S2: Missing data assessment Stan code and algorithm details.
