# Supplementary material for: Changes in Vertical Stratification of Neotropical Nymphalid Butterflies at Forest Edges Are Not Directly Caused by Light and Temperature Conditions
Source: Insects. 2025 Jan 11;16(1):64. doi: 10.3390/insects16010064 (PMC11765654; doi:10.3390/insects16010064)
Supplement: Supplementary file 1 [file insects-16-00064-s001.zip › insects-3325667-supplementary.pdf]

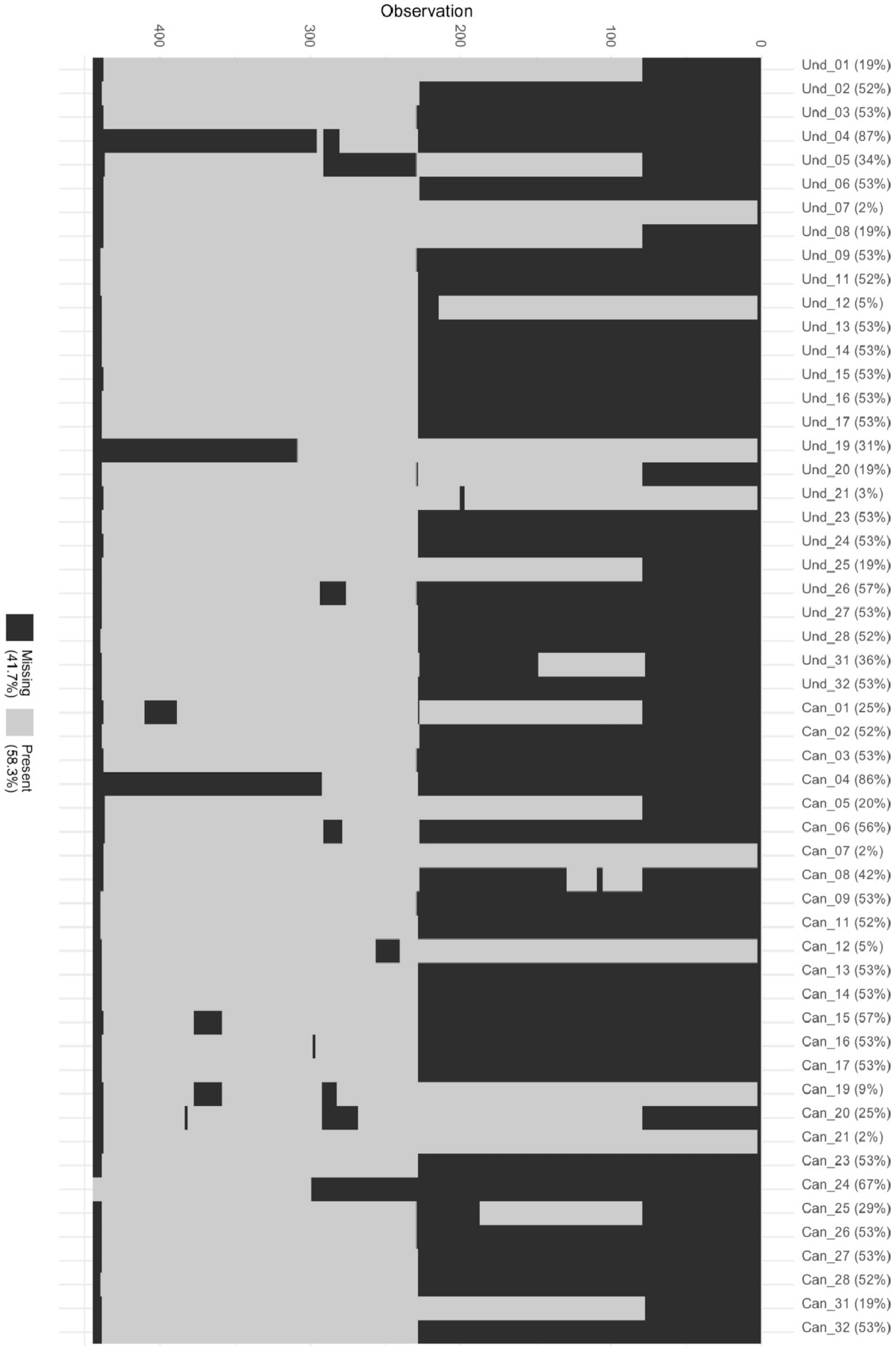

Figure S1. Missingness map for logger locations.

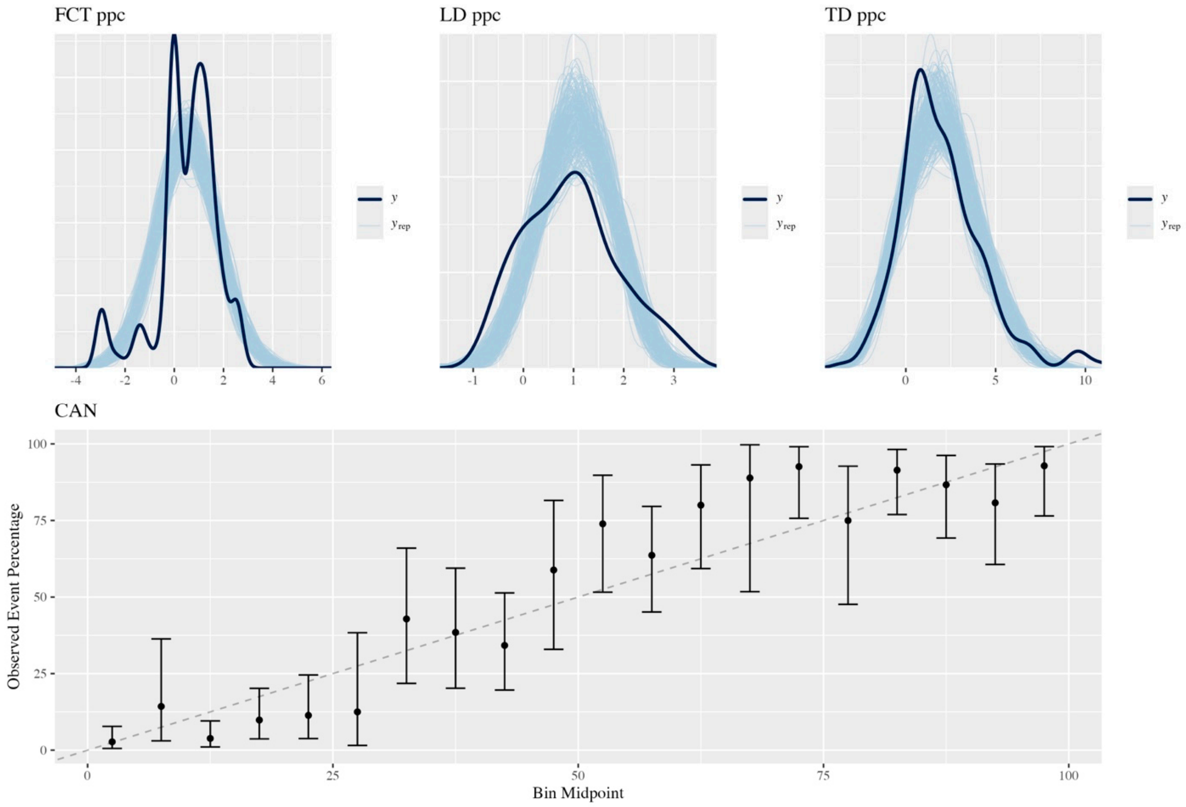

Figure S2. Posterior predictive checks comparing density estimates for observed data and predictive distribution samples for Forest Canopy Tendency (FCT), Log Light Difference (LD), and Temperature Difference (TD) regressions. Calibration plot for CAN (bottom) compares binned CAN observations to model expected values for CAN.

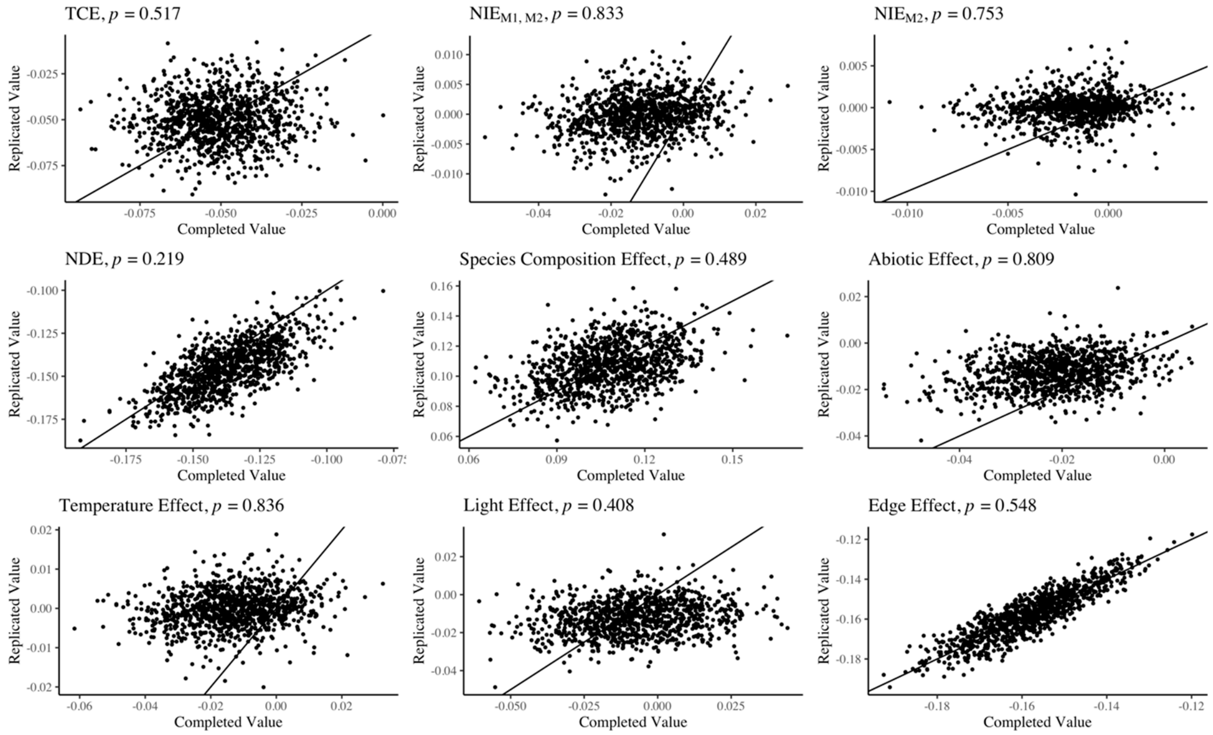

Figure S3. Posterior predictive checks and posterior predictive p-values (1000 replicates) for missing data model assessment.

For all temperature and log illuminance time series plots, canopy values are in cyan, and understory values are in orange. Solid lines indicate posterior medians, and shading indicates 95% credible intervals. Edge locations were traps 1-10, ridge locations were traps 11, 12, 17, 21-26, 28-30, and valley locations were traps 13-16, 18-20, 27, 31, 32.

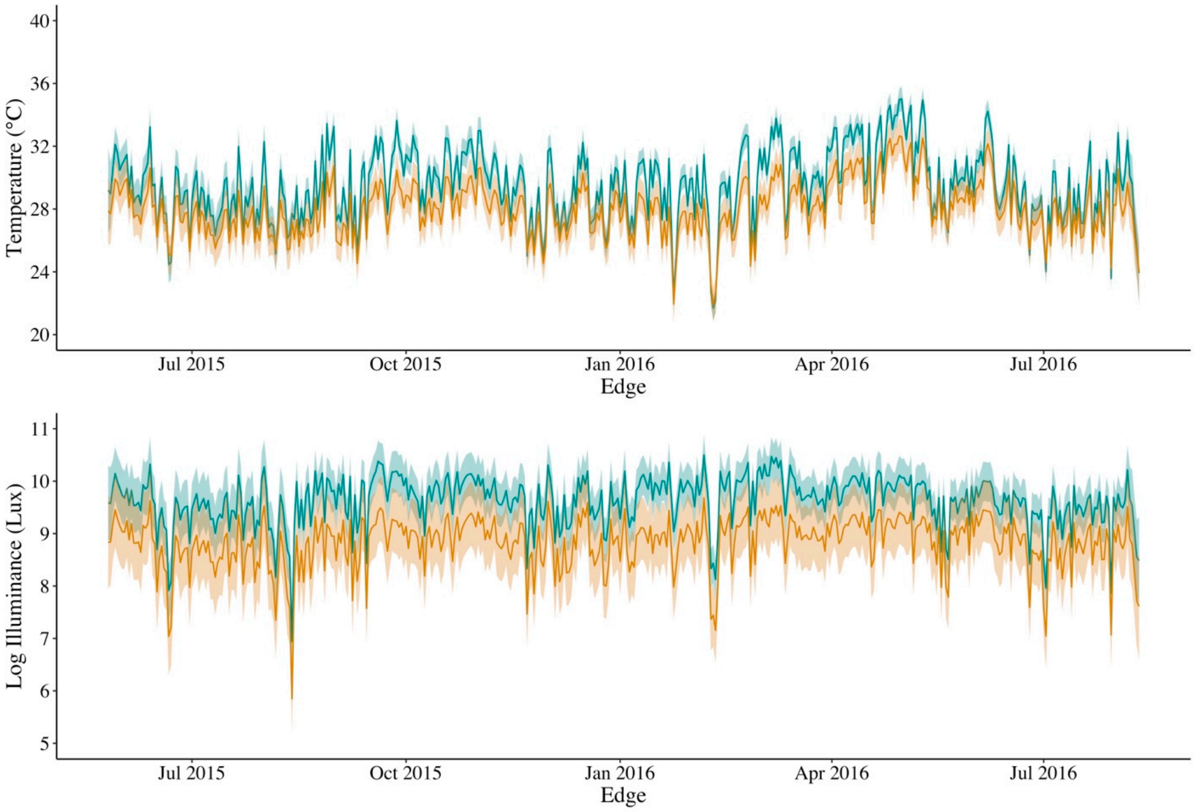

Figure S4. Temperature and log illuminance state estimates for edge habitat.

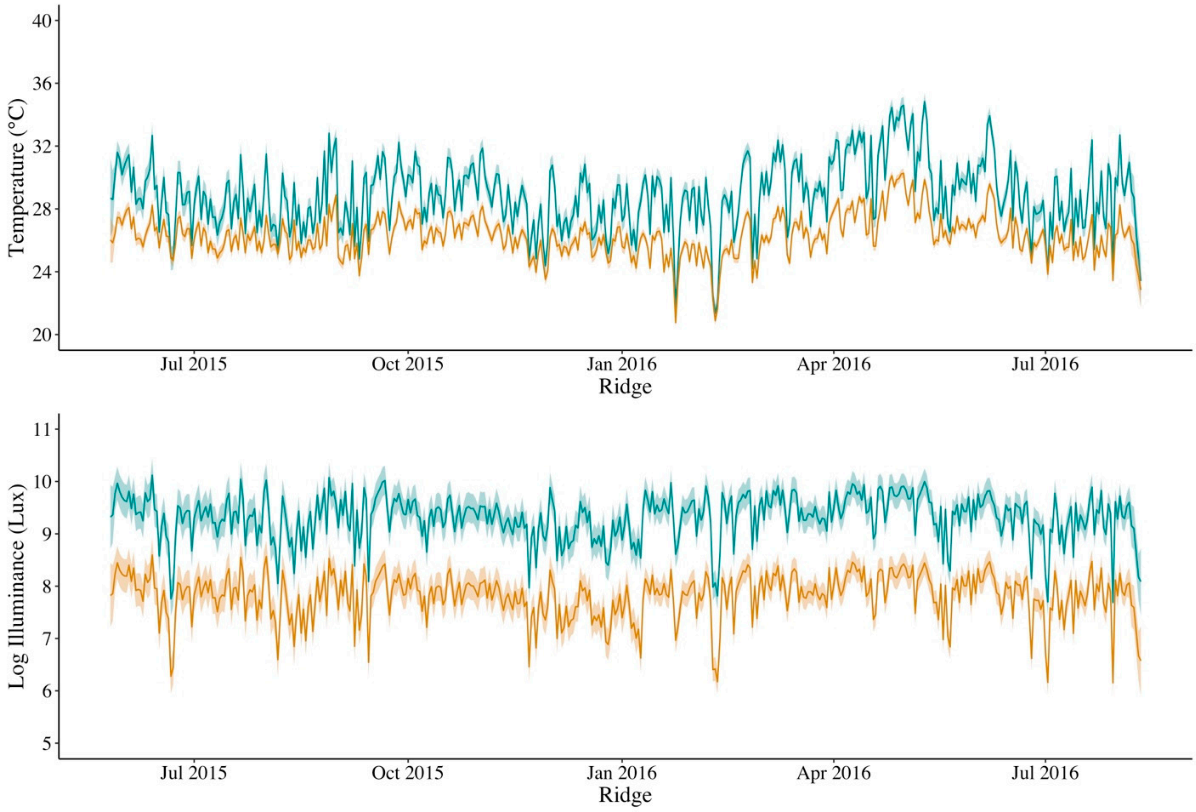

Figure S5. Temperature and log illuminance state estimates for ridge habitat.

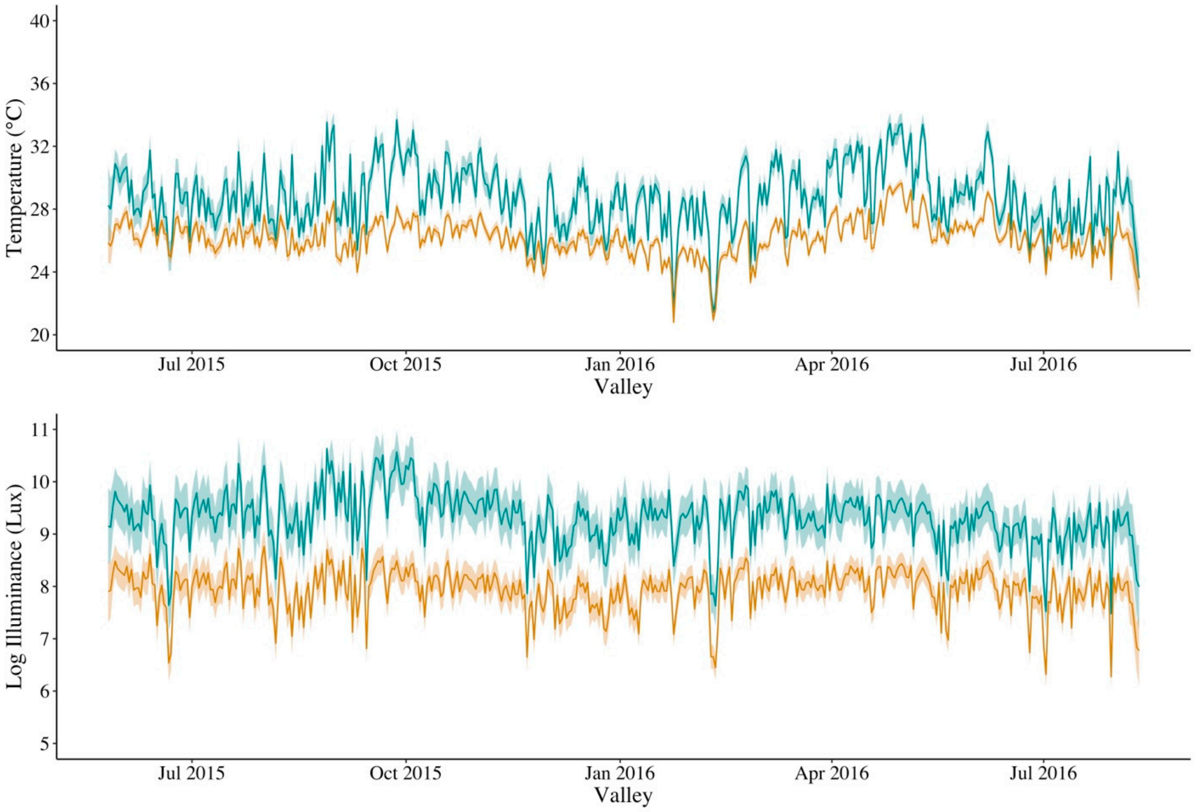

Figure S6. Temperature and log illuminance state estimates for valley habitat.

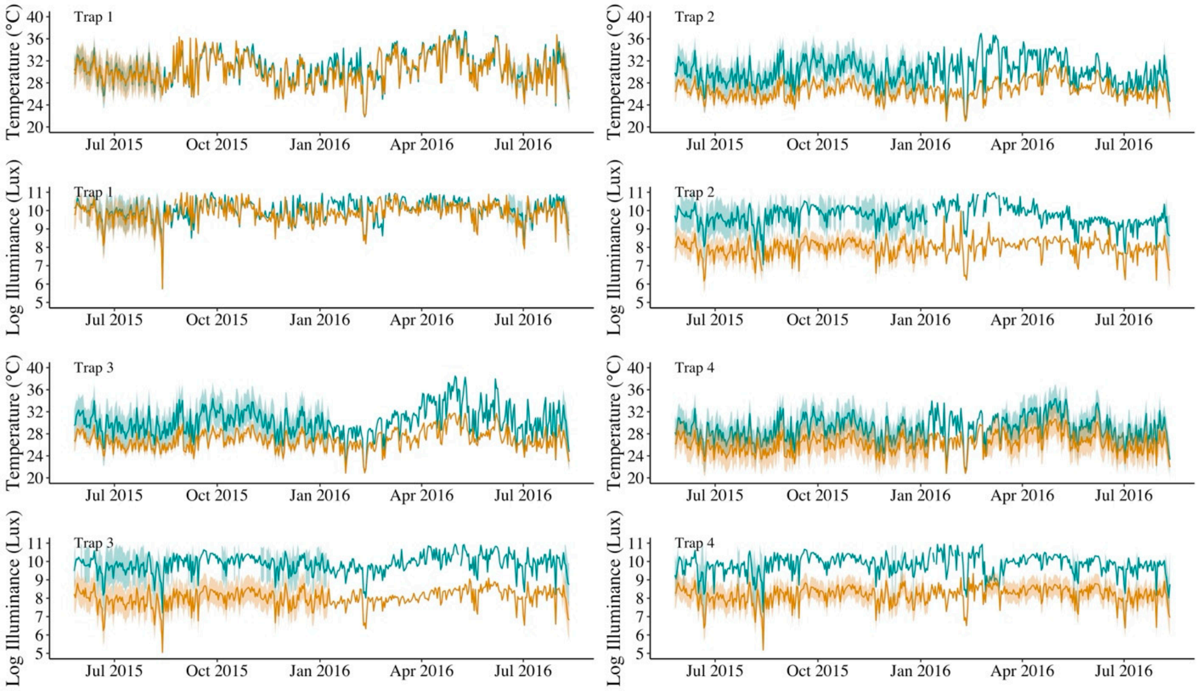

Figure S7. Temperature and log illuminance series for traps 1-4.

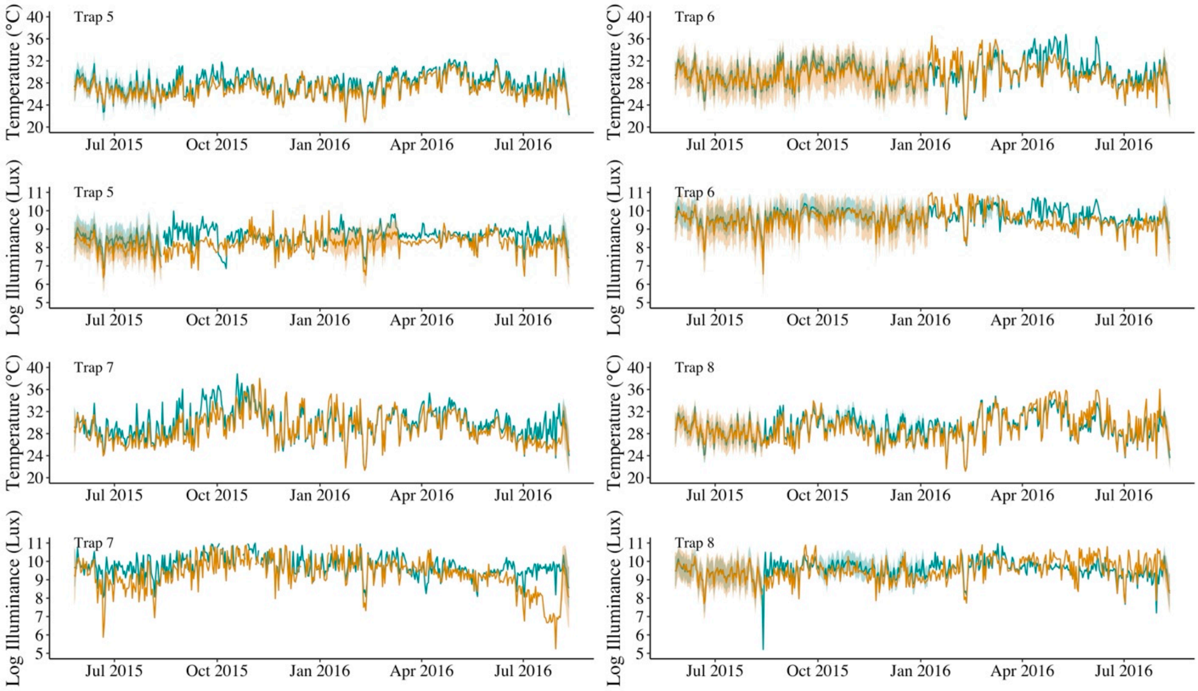

Figure S8. Temperature and log illuminance series for traps 5-8.

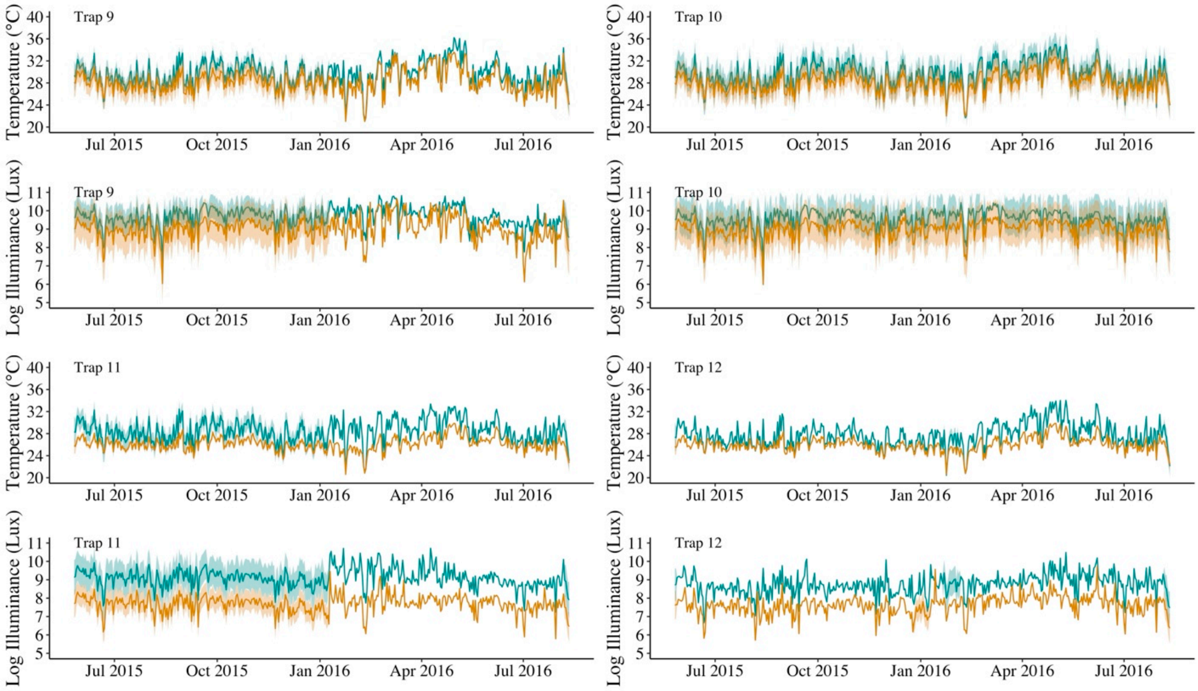

Figure S9. Temperature and log illuminance series for traps 9-12.

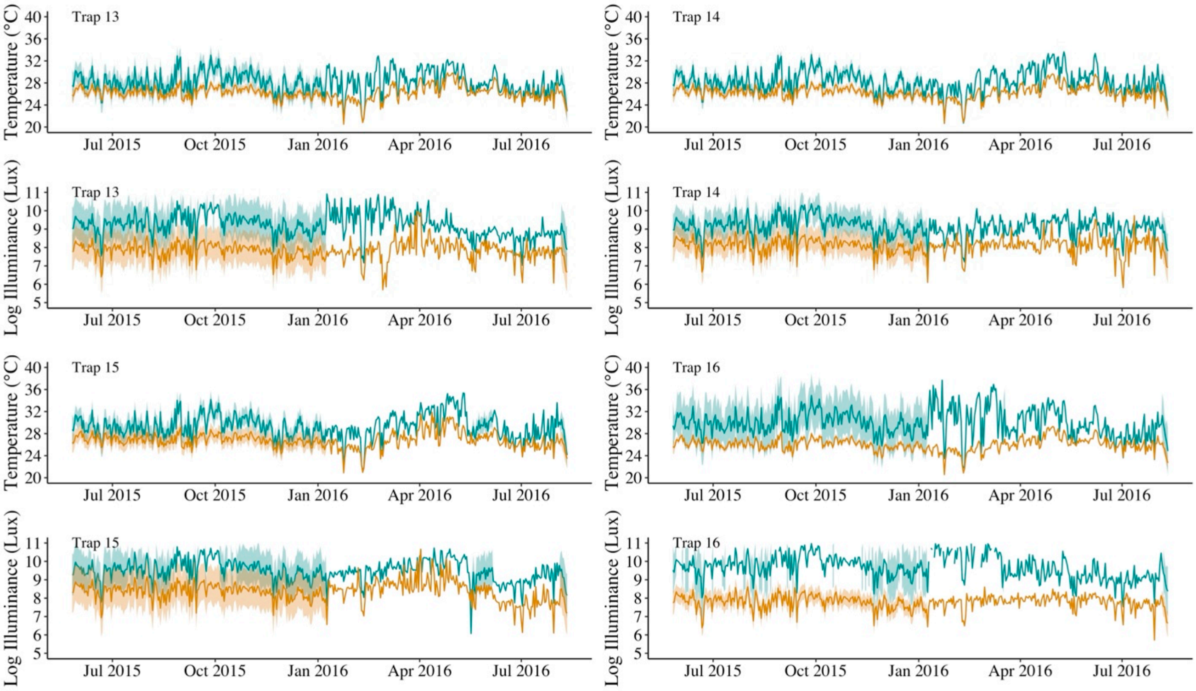

Figure S10. Temperature and log illuminance series for traps 13-16.

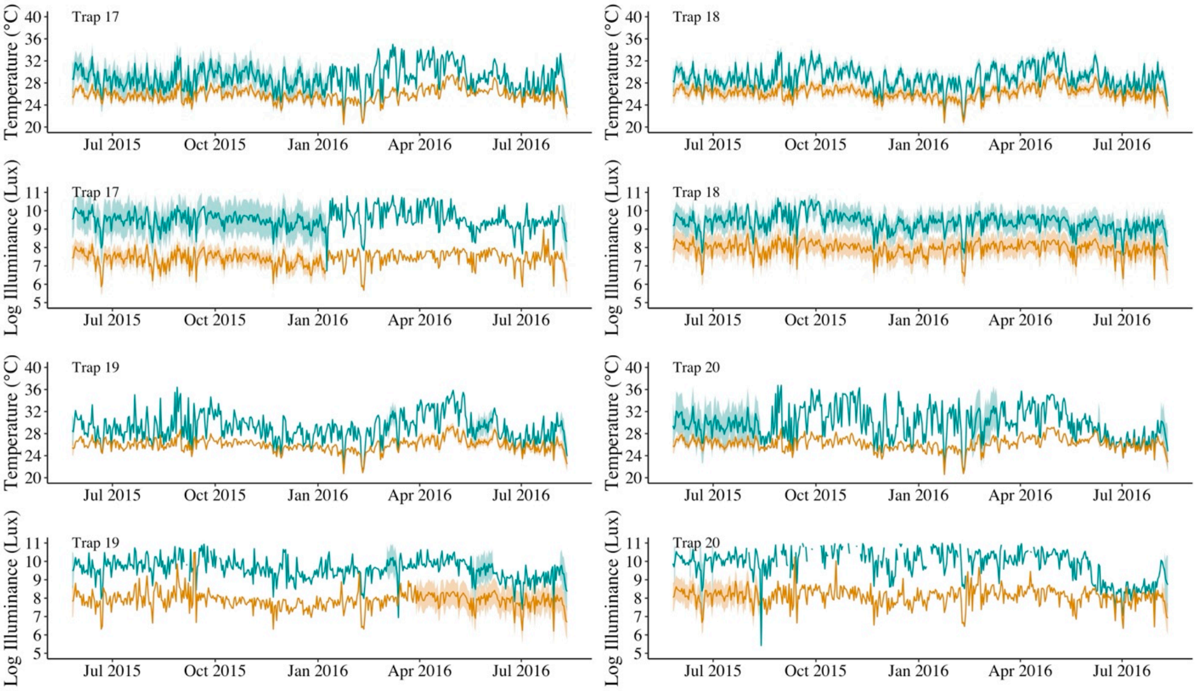

Figure S11. Temperature and log illuminance series for traps 17-20.

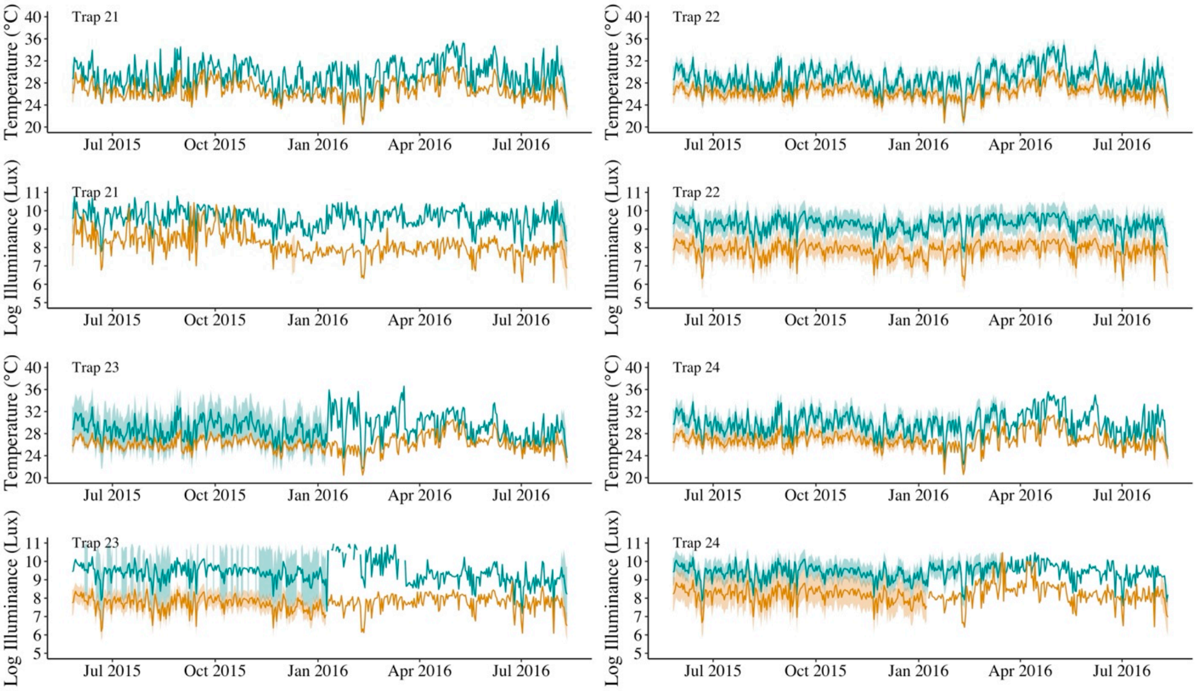

Figure S12. Temperature and log illuminance series for traps 21-24.

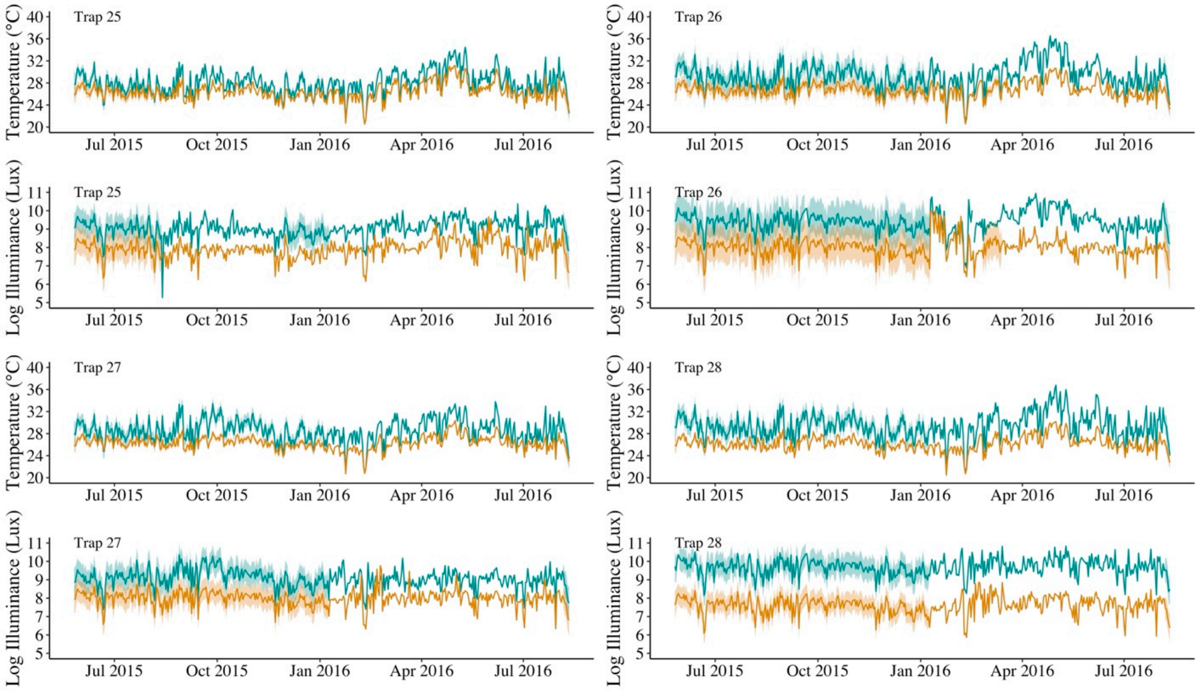

Figure S13. Temperature and log illuminance series for traps 25-28.

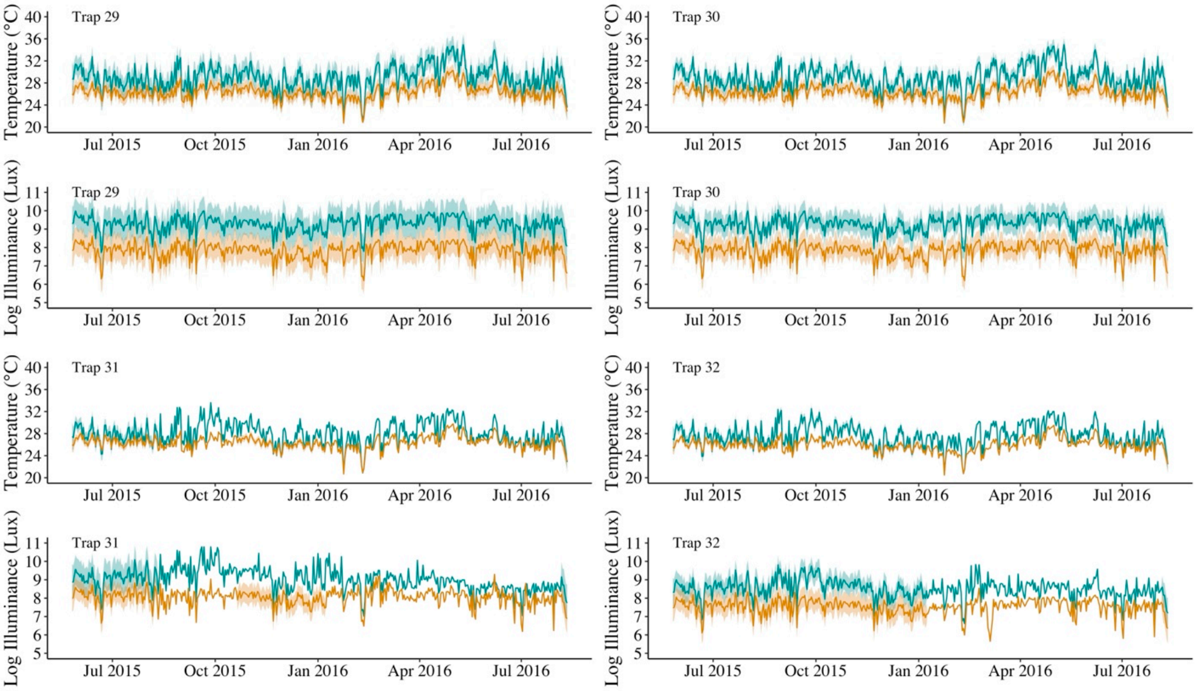

Figure S14. Temperature and log illuminance series for traps 29-32.

Table S1. Butterfly capture counts for edge canopy (EC), edge understory (EU), valley canopy (VC), valley understory (VU), ridge canopy (RC), and ridge understory (RU) locations.

			Forest			
Species	EC	EU	VC	VU	RC	RU
Adelpha cytherea	0	1	0	0	0	0
Adelpha iphiclus	5	6	6	0	1	0
Adelpha naxia	1	0	0	0	1	0
Anartia fatima	0	2	0	0	0	0
Anartia jatrophae	0	0	0	0	1	0
Antirrhea philaretes	0	0	0	17	1	5
Archaeoprepona camilla	0	0	0	1	0	2
Archaeoprepona demophon	2	14	1	3	0	0
Archaeoprepona demophoon	1	0	0	0	2	0
Archaeoprepona meander	0	2	0	1	0	0
Caerois gerdrudtus	0	0	0	1	0	0
Caligo atreus	0	4	0	12	0	8
Caligo brasiliensis	0	9	0	9	0	3
Caligo illioneus	0	3	0	0	0	0
Caligo oedipus	0	1	0	0	0	0
Caligo telamonius	0	1	0	0	0	0
Callicore astarte	0	0	7	1	3	0
Callicore lyca	1	0	0	0	0	0
Callicore texa	0	0	1	0	0	0
Catagramma tolima	0	0	1	0	0	0
Catoblepia orgetorix	0	2	0	7	1	17
Catoblepia xanthicles	0	0	0	0	1	0
Catonephele mexicana	1	1	0	0	0	0
Catonephele numilia	4	17	1	1	3	1
Catonephele orites	1	13	7	2	7	10
Chloreuptychia arnaca	0	0	0	4	0	1
Cissia confusa	1	1	2	0	1	2
Cissia pompilia	0	6	0	0	0	0
Cissia pseudoconfusa	1	3	0	0	0	0
Cithaerias pireta	0	0	0	4	1	6
Colobura annulata	1	1	4	2	1	0
Colobura dirce	0	2	0	0	0	0
Consul panariste	0	0	0	1	0	0
Diaethria clymena	4	1	0	0	0	0
Dryas iulia	0	4	1	0	0	0
Dulcedo polita	0	2	1	18	0	8
Dynamine postverta	1	0	0	0	0	0
Epiphile adrasta	0	1	1	0	0	0
Eryphanis lycomedon	0	7	0	0	0	2
Fountainea eurypyle	0	1	0	0	1	0
Hamadryas amphinome	4	0	1	0	3	0
Hamadryas arinome	1	1	3	1	7	2
Hamadryas feronia	1	1	0	0	0	0
Hamadryas laodamia	9	1	2	0	10	0

Species	EC	EU	VC	Forest VU	RC	RU
Heliconius cydno	0	0	0	0	0	1
Heliconius hecale	1	0	0	0	0	0
Hermeuptychia intricata/hermes	9	14	0	0	0	0
Historis acheronta	0	1	0	0	0	0
Historis odius	2	2	0	0	2	0
Magneuptychia gomezi	1	1	0	0	2	1
Marpesia berania	0	1	0	0	0	0
Marpesia chiron	0	1	0	0	0	0
Marpesia merops	0	0	0	1	0	0
Megeuptychia antonoe	1	0	0	1	0	0
Memphis artacaena	1	1	0	0	2	0
Memphis aureola	1	0	0	0	0	0
Memphis cleomestra	1	0	1	0	1	1
Memphis glauce	0	0	3	0	3	0
Memphis mora	0	1	1	0	0	0
Memphis moruus	11	3	1	0	2	0
Memphis oenomais	0	1	0	0	0	0
Memphis philumena	1	4	0	0	0	0
Memphis pithyusa	0	0	0	0	1	0
Memphis proserpina	0	0	1	0	3	0
Morpho deidamia	0	0	0	7	0	0
Morpho helenor	0	0	0	17	0	2
Myscelia cyaniris	3	14	0	0	0	1
Myscelia leucocyana	6	2	2	0	3	0
Nessaea aglaura	0	5	5	11	2	18
Nica flavilla	1	0	1	0	0	0
Opsiphanes bogotanus	0	0	0	0	1	0
Opsiphanes cassina	6	0	1	0	3	1
Opsiphanes invirae	0	0	0	0	0	1
Opsiphanes quiteria	0	1	0	0	0	0
Opsiphanes tamarindi	0	0	2	0	0	0
Pareuptychia metaleuca	0	5	1	1	0	2
Pierella helvina	0	0	0	3	0	1
Prepona aedon	0	0	1	0	0	0
Prepona gnorima	0	0	0	0	1	0
Prepona laertes	16	2	4	0	3	0
Pseudodebis zimri	0	1	0	0	0	1
Pyrrhogyra neaerea	0	1	2	0	3	1
Pyrrhogyra otolais	1	1	0	0	2	0
Siderone galanthis	0	0	0	0	3	0
Smyrna blomfildia	1	0	0	0	0	0
Taygetis thamyra	0	15	0	3	0	2
Taygetis virgilia	0	1	0	0	0	0
Temenis laothoe	3	1	3	2	4	0
Tigridia acesta	0	2	1	0	0	2

Species	Forest					
	EC	EU	VC	VU	RC	RU
Zaretis ellops	0	0	1	0	0	0
Zaretis isidora	0	2	2	0	3	3
Zaretis itys	0	3	5	5	2	2

Equations S1-S28. Main model equations. Design matrix A matches trap series to their habitat-stratum state estimates.

For day *t*,

$$v_t \sim MVN(\overline{v} + Aq_t, \Sigma_v). \tag{S1}$$

$$q_t \sim MVN(Cq_{t-1}, \Sigma_q). \tag{S2}$$

$$C = \begin{bmatrix} \phi_1 & \cdots & 0 \\ \vdots & \ddots & \vdots \\ 0 & \cdots & \phi_{12} \end{bmatrix}. \tag{S3}$$

$$\phi_1 \dots \phi_{12} \sim U(-1{,}1). \tag{S4}$$

$$\Sigma_v = \begin{bmatrix} \sigma_{v,1}^2 & \cdots & 0 \\ \vdots & \ddots & \vdots \\ 0 & \cdots & \sigma_{v,108}^2 \end{bmatrix}. \tag{S5}$$

$$\Sigma_q = \begin{bmatrix} \sigma_{q,1} & \cdots & 0 \\ \vdots & \ddots & \vdots \\ 0 & \cdots & \sigma_{q,12} \end{bmatrix} \Omega \begin{bmatrix} \sigma_{q,1} & \cdots & 0 \\ \vdots & \ddots & \vdots \\ 0 & \cdots & \sigma_{q,12} \end{bmatrix}. \tag{S6}$$

$$\sigma_v \sim MVN\left(A\theta,\ \mathrm{diag}(A\sigma_{\sigma,v})\right). \tag{S7}$$

$$\Omega \sim LKJ(2). \tag{S8}$$

$$\sigma_{q,1} \dots \sigma_{q,12} \sim N_{+}(0{,}5) \tag{S9}$$

$$\overline{v} \sim MVN\left(A\eta,\ \mathrm{diag}\left(A(\sigma_{\eta,1} \dots \sigma_{\eta,12})\right)\right). \tag{S10}$$

$$\overline{y} \sim MVN\left(A\mu,\ \mathrm{diag}(A\sigma_\eta^2)\right). \tag{S11}$$

$$\sigma_{\eta,1} \dots \sigma_{\eta,12} \sim N_{+}(0{,}5). \tag{S12}$$

$$\eta \sim MVN\left(\begin{bmatrix} 26 \\ \vdots \\ 2 \end{bmatrix}, \begin{bmatrix} 25 & \cdots & 0 \\ \vdots & \ddots & \vdots \\ 0 & \cdots & 36 \end{bmatrix}\right). \tag{S13}$$

$$\begin{bmatrix} \sigma_{v,1} \\ \vdots \\ \sigma_{v,108} \end{bmatrix} \sim MVN\left(A\begin{bmatrix} \theta_1 \\ \vdots \\ \theta_{12} \end{bmatrix},\ A\begin{bmatrix} \sigma_{\sigma v,1}^2 \\ \vdots \\ \sigma_{\sigma v,12}^2 \end{bmatrix}\right). \tag{S14}$$

$$\theta_1 \dots \theta_{12} \sim N_{+}(0{,}5). \tag{S15}$$

$$\sigma_{\sigma v,1} \dots \sigma_{\sigma v,12} \sim N_{+}(0{,}5). \tag{S16}$$

For missing trap *i*,

$$\widehat{v_\iota} = q_i + \overline{v}_{mis,i} + \sigma_{\hat{v},i} \times \tilde{\hat{v}}_\iota. \tag{S17}$$

$$\overline{v}_{mis,i} = \eta_i + \sigma_{\eta_{,i}} \times \tilde{\overline{v}}_{mis,i}. \tag{S18}$$

$$\sigma_{\hat{v},i} \sim N(\theta_i, \sigma_{\sigma,v_i}). \tag{S19}$$

$$\tilde{\hat{v}}_i \sim N(0{,}1). \tag{S20}$$

$$\tilde{\tilde{v}}_{mis,i} \sim N(0,1). \tag{S21}$$

For species *s* from tribe *j*,

$$FCT_{obs,s,j} \sim N\left(FCT_{s,j}, \sigma_{FCT,s,j}\right). \tag{S22}$$

$$FCT_{s,j} = \gamma_j + \xi_j \widetilde{FCT}_{s,j}. \tag{S23}$$

$$\widetilde{FCT}_{s,j} \sim N(0,1), \quad \gamma_j \sim N(0,2.5), \quad \text{and} \quad \xi_j \sim N_+(0,2.5). \tag{S24}$$

For individual butterfly *n*,

$$LD_n \sim N(\alpha_1 + \beta_1 FE_n,\ \tau_1). \tag{S25}$$

$$TD_n \sim N(\alpha_3 + \beta_3 FE_n + \beta_4 LD_n,\ \tau_3). \tag{S26}$$

$$FCT_{n,s,j} \sim N(\alpha_2 + \beta_2 FE_n,\ \tau_2). \tag{S27}$$

$$\Pr(CAN_n = 1) = \text{inv logit}\left(\alpha_4 + \beta_5 FE_n + \beta_6 LD_n + \beta_7 TD_n + \beta_8 FCT_{n,s,j}\right). \tag{S28}$$

Equations S29-S37. Estimated causal effects.

$$\text{Light Effect} = E\left\{CAN\left(1, LD(1), TD\big(0, LD(0)\big), FCT(0)\right)\right\} - E\left\{CAN\left(1, (0), TD\big(0, LD(0)\big), FCT(0)\right)\right\}. \quad \text{(S29)}$$

$$\text{Temperature Effect} = E\left\{Y\left(1, M_1(1), M_2\big(1, M_1(1)\big), Z(0)\right)\right\} - E\left\{Y\left(1, M_1(1), M_2\big(0, M_1(0)\big), Z(0)\right)\right\}. \quad \text{(S30)}$$

$$\text{Species Composition Effect} = E\left\{CAN\left(1, LD(1), TD\big(1, LD(1)\big), FCT(1)\right)\right\} - E\left\{CAN\left(1, LD(1), TD\big(1, LD(1)\big), FCT(0)\right)\right\}. \quad \text{(S31)}$$

$$NIE_{M1,M2} = E\left\{Y\left(CAN, LD(1), TD\big(1, LD(1)\big), FCT(0)\right)\right\} - E\left\{CAN\left(1, LD(1), TD\big(1, LD(0)\big), FCT(0)\right)\right\}. \quad \text{(S32)}$$

$$NIE_{M2} = E\left\{CAN\left(1, LD(1), TD\big(1, LD(0)\big), FCT(0)\right)\right\} - E\left\{CAN\left(1, LD(1), TD\big(0, LD(0)\big), FCT(0)\right)\right\}. \quad \text{(S33)}$$

$$NDE = E\left\{CAN\left(1, LD(0), TD\big(0, LD(0)\big), FCT(0)\right)\right\} - E\left\{CAN\left(0, LD(0), TD\big(0, LD(0)\big), FCT(0)\right)\right\}. \quad \text{(S34)}$$

$$TCE = NIE_{M1,M2} + \text{Light Effect} + NIE_{M2} + NDE + \text{Species Composition Effect}. \quad \text{(S35)}$$

$$\text{Abiotic Effect} = NIE_{M1,M2} + \text{Light Effect} + NIE_{M2}. \quad \text{(S36)}$$

$$\text{Temperature Effect} = NIE_{M1,M2} + NIE_{M2}. \quad \text{(S37)}$$

Code S1. Main analysis model Stan code and algorithm details.

We used four chains with 2000 warmup and 10000 sampling iterations for each chain (thinned by a factor of five) with treedepth = 11 and adapt delta = 0.99. CmdStan's diagnose utility showed no issues with E-BFMI, divergent transitions, effective sample size, or split R-hat (though treedepth showed inefficient trajectories). All parameters had > 400 effective sample size and < 1.02 R-hat as calculated using CmdStan's stansummary utility.

```
data {
// abiotic time series
	int T;
	int Num_st;
	int K;
	int total_mis;
	int total_obs;
	array [K] int start_mis;
	array [K] int start_obs;
	array [K] int end_mis;
	array [K] int end_obs;
	array [total_mis] int place_mis;
	array [total_obs] int place_obs;
	row_vector [total_obs] v_obs;
	matrix [K, Num_st] A; // Design matrix
	vector [K] vbar;

// reshaping abiotic data
	array [K] int col_IDs_new;
	array [K] int not_empty;
	array [20] int empty;

// canopy preferences
	int Num_tr; //
	int S_obs;
	int S_mis;
	vector [S_obs] FCT_obs; //
	vector <lower=0> [S_obs] sd_FCT_obs; //
	array [S_obs] int ID_obs;
	array [Num_tr] int sp_index; // number of species per tribe

// mediation model
	int N;
	array [N] int CAN; // canopy observation
	array [N] int species_ID;
	array [N] int day_ID;
	array [N] int trap;
	vector [N] FE; // edge, binary, but must be vector for dat
}
transformed data {
	int S = S_obs + S_mis;
```

```
	array [20] int u = {1,2,7,8,5,6,11,12,3,4,9,10,3,4,9,10,3,4,9,10};
}
parameters {
// abiotic time series
	row_vector [total_mis] v_mis;
	array [T] vector [Num_st] q;
	vector [Num_st] eta;
	vector <lower=0> [Num_st] sig_eta;
	vector <lower=0> [Num_st] sig_q;
	vector <lower=0> [K] sig_v;
	vector <lower=0> [Num_st] sigsig_v;
	vector <lower=0> [Num_st] theta;
	cholesky_factor_corr [Num_st] L_Omega;
	vector <lower=-1, upper=1> [Num_st] phi_v;
	matrix [20, T] vhat_tilde;
	vector [20] vbar_mis_tilde;
	vector <lower=0> [20] sig_vhat;

// canopy preference model
	vector [S] FCT_tilde;
	vector <lower=0> [Num_tr] xi; // tribe sds
	vector [Num_tr] gamma; // tribe means

// mediation model
	vector [4] a; // intercepts for LD, TD, FCT, and CAN
	vector [8] b; // 1 for LD, 2:3 for TD, 4 for FCT, 5:8 for CAN
	vector <lower=0> [3] tau; // LD, TD, FCT
}
transformed parameters {
	matrix [K, T] v;
	vector [S] FCT; // forest canopy tendencies
	matrix [20, T] vhat;
	vector [20] vbar_mis;
	matrix [N, 4] dat; // FE, LD, TD, FCT

	for (k in 1:K) {
		v[k,place_mis[start_mis[k]:end_mis[k]]] = v_mis[start_mis[k]:end_mis[k]];
		v[k,place_obs[start_obs[k]:end_obs[k]]] = v_obs[start_obs[k]:end_obs[k]];
	}

	{
	int pos = 1;
	for (j in 1:Num_tr) {
		FCT[pos:(pos + sp_index[j]) - 1] =
			gamma[j] + xi[j]*FCT_tilde[pos:(pos + sp_index[j]) - 1];
		pos = pos + sp_index[j];
		}
	}
```

```
// unobserved locations
// 10 edge, 18 valley, 22 ridge, 29 ridge, 30 ridge

  vbar_mis = eta[u] + sig_eta[u].*vbar_mis_tilde;
  for (i in 1:20) {
    vhat[i,] = to_row_vector(q[,u[i]]) + vbar_mis[i] + sig_vhat[i]*vhat_tilde[i,];
  }

// preparing dat

        {
                matrix [T, 128] new_mat; // T_u, T_c, LL_u, LL_c
                matrix [32*T, 2] two_col;

                for (k in 1:K) {
                        new_mat[,not_empty[k]] = v[col_IDs_new[k],]';
                }
                for (i in 1:20) {
                        new_mat[, empty[i]] = vhat[i,]';
                }

                for (i in 1:32) {
                        two_col[(T*i-T+1):(T*i),1] = new_mat[,(4*i-2)]-new_mat[,(4*i-3)];
                        two_col[(T*i-T+1):(T*i),2] = new_mat[,(4*i)]-new_mat[,(4*i-1)];
                }
// dat columns FE, LD, TD, FCT.
                dat[, 1] = FE;
                for (n in 1:N) {
                        dat[n, 2] = two_col[day_ID[n] + T*(trap[n]-1), 2];
                        dat[n, 3] = two_col[day_ID[n] + T*(trap[n]-1), 1];
                        dat[n, 4] = FCT[species_ID[n]];
                }
        }
        // to check
        //      print("dat check: ", dat[167, 1:3]); // should be [0,1.83492,2.22655]
}
model {
// abiotic series
        array [T] vector [Num_st] q_mu;
        matrix [K, T] v_mu;
        matrix [Num_st, Num_st] C = diag_matrix(phi_v);
        matrix [Num_st, Num_st] L_cov = diag_pre_multiply(sig_q, L_Omega);

        q_mu[1, ] = to_vector(rep_array(0.0, Num_st));
        for (t in 2:T) {
                q_mu[t,] = C*q[t-1, ];
        }
```

```
		L_Omega ~ lkj_corr_cholesky(2);
		sig_q ~ normal(0, 5);
		sig_eta ~ normal(0, 5);
		theta ~ normal(0, 5);
		sig_v ~ normal(A*theta, A*sigsig_v);
		sigsig_v ~ normal(0, 5);

		sig_vhat ~ normal(theta[u], sigsig_v[u]);

		to_vector(vhat_tilde) ~ std_normal();
		vbar_mis_tilde ~ std_normal();

		eta[1:6] ~ normal(26, 5);
		eta[7:12] ~ normal(2, 6);

		vbar ~ normal(A*eta, A*sig_eta);

		q ~ multi_normal_cholesky(q_mu, L_cov);

		for (t in 1:T) {
			v_mu[,t] = vbar + A*q[t,];
		}
		for (k in 1:K) {
			v[k] ~ normal(v_mu[k], sig_v[k]);
		}

// canopy preference model
		FCT_tilde ~ std_normal();
		gamma ~ normal(0, 2.5);
		xi ~ normal(0, 2.5);
		FCT_obs ~ normal(FCT[ID_obs], sd_FCT_obs);

// mediation model
		dat[,2] ~ normal(a[1] + FE*b[1], tau[1]); // LD ~ FE
		dat[,3] ~ normal(a[2] + FE*b[2] + dat[,2]*b[3], tau[2]); // TD ~ FE + LD
		dat[,4] ~ normal(a[3] + FE*b[4], tau[3]); // FCT ~ FE
		CAN ~ bernoulli_logit_glm(dat, a[4], b[5:8]); // CAN ~ FE + TD + LD + FCT
}
generated quantities {
// abiotic time series
		array [T] vector [Num_st] x;
		matrix [Num_st, Num_st] Omega = multiply_lower_tri_self_transpose(L_Omega);
  matrix [K, T] vrep;
  matrix [20, T] vhatrep;

		real NIE_Z; // Species Composition Effect
		real NDE;
```

```
	real NIE_2;
	real NIE_12;
	real TCE;
	real abiotic_effect;
	real temp_effect;
	real light_effect;
	real edge_effect;
	matrix [2, 2] abiotic_fit; // LD and TD fitted values for forest and edge.
	matrix [S, 2] fit; // forest and edge canopy probability estimates for species.
	vector [N] fitted;
	vector [N] FCTrep;
	vector [N] LDrep;
	vector [N] TDrep;

	for (t in 1:T) {
		x[t] = q[t] + eta;
	}

// mediation model
{
	matrix [N, 2] LD_pred; // LD(FE = 0), LD(FE = 1)
	matrix [N, 3] TD_pred; // 00, 10, 11
	matrix [N, 2] FCT_pred; // FCT(FE = 0), FCT(FE = 1)
	matrix [N, 6] can_est; // FE, LD, TD(, LD()), FCT  00000, 10000 11000, 11100, 11110, 11111

// dat columns FE, LD, TD, FCT.
	for (n in 1:N) {
		if (FE[n] == 0) {
			LD_pred[n, 1] = dat[n, 2];
			LD_pred[n, 2] = normal_rng(a[1] + b[1], tau[1]);
			TD_pred[n, 1] = dat[n, 3];
			TD_pred[n, 3] = normal_rng(a[2] + b[2] + LD_pred[n, 2]*b[3], tau[2]);
			FCT_pred[n ,1] = dat[n, 4];
			FCT_pred[n, 2] = normal_rng(a[3] + b[4],  tau[3]);
		} else {
			LD_pred[n, 1] = normal_rng(a[1], tau[1]);
			LD_pred[n, 2] = dat[n, 2];
			TD_pred[n, 1] = normal_rng(a[3] + LD_pred[n, 1]*b[3], tau[2]);
			TD_pred[n, 3] = dat[n, 3];
			FCT_pred[n, 1] = normal_rng(a[3], tau[3]);
			FCT_pred[n, 2] = dat[n, 4];
		}
		TD_pred[n, 2] = normal_rng(a[2] + b[2] + LD_pred[n, 1]*b[3], tau[2]);
	}

	can_est[, 1] =
		inv_logit(a[4] + LD_pred[, 1]*b[6] + TD_pred[, 1]*b[7] + FCT_pred[, 1]*b[8]);
	can_est[, 2] =
```

```
			inv_logit(a[4] + b[5] + LD_pred[, 1]*b[6] + TD_pred[, 1]*b[7] + FCT_pred[, 1]*b[8]);
		can_est[, 3] =
			inv_logit(a[4] + b[5] + LD_pred[, 2]*b[6] + TD_pred[, 1]*b[7] + FCT_pred[, 1]*b[8]);
		can_est[, 4] =
			inv_logit(a[4] + b[5] + LD_pred[, 2]*b[6] + TD_pred[, 2]*b[7] + FCT_pred[, 1]*b[8]);
		can_est[, 5] =
			inv_logit(a[4] + b[5] + LD_pred[, 2]*b[6] + TD_pred[, 3]*b[7] + FCT_pred[, 1]*b[8]);
		can_est[, 6] =
			inv_logit(a[4] + b[5] + LD_pred[, 2]*b[6] + TD_pred[, 3]*b[7] + FCT_pred[, 2]*b[8]);

		abiotic_fit[1, 1] = normal_rng(a[1], tau[1]); // LD forest
		abiotic_fit[1, 2] = normal_rng(a[1] + b[1], tau[1]); // LD edge
		abiotic_fit[2, 1] = normal_rng(a[2] + abiotic_fit[1, 1]*b[3], tau[2]); // TD forest
		abiotic_fit[2, 2] = normal_rng(a[2] + b[2] + abiotic_fit[1, 2]*b[3], tau[2]); // TD edge

		fit[, 1] = inv_logit(a[4] + abiotic_fit[1, 1]*b[6] +  abiotic_fit[2, 1]*b[7] + FCT*b[8]); // forest
		fit[, 2] = inv_logit(a[4] + b[5] + abiotic_fit[1, 2]*b[6] +  abiotic_fit[2, 2]*b[7] + FCT*b[8]); // edge

		NDE = mean(can_est[, 2] - can_est[, 1]);
		NIE_2 = mean(can_est[, 4] - can_est[, 3]);
		NIE_12 = mean(can_est[, 5] - can_est[, 4]);
		NIE_Z = mean(can_est[, 6] - can_est[, 5]);
		light_effect = mean(can_est[, 3] - can_est[, 2]); // same as NIE_1
		temp_effect = NIE_2 + NIE_12;
		abiotic_effect = light_effect + temp_effect;
		TCE = NDE + light_effect + abiotic_effect + NIE_Z; //
		edge_effect = TCE - NIE_Z;


  for (n in 1:N) {
    LDrep[n] = normal_rng(a[1] + dat[n, 1]*b[1], tau[1]);
    TDrep[n] = normal_rng(a[2] + dat[n, 1]*b[2] + dat[n, 2]*b[3], tau[2]);
    FCTrep[n] = normal_rng(a[3] + dat[n, 1]*b[4], tau[3]);
  }
  fitted = inv_logit(a[4] + dat*b[5:8]);
}

// Calculations for posterior replications.

    for (t in 1:T) {
			vrep[,t] = to_vector(normal_rng(vbar + A*q[t,], sig_v));
    }
    for (i in 1:20) {
      vhatrep[i,] = to_row_vector(q[,u[i]]) + vbar_mis[i] + sig_vhat[i]*vhat_tilde[i,];
		}
}
```

Code S2. Missing data assessment Stan code and algorithm details.
Model fitting was done using Stan's optimization algorithm; a maximum of 5000 iterations each were used for completed and fully replicated data model fitting.

```
data {
	int N;
	int nreps;
	int CAN [N]; // canopy observation
	array [nreps] matrix [N, 4] dat; //  FE, LD, TD, FCT
}
transformed data {
	vector [N] FE = dat[1,,1];
}
parameters {
	array [nreps] vector [4] a;
	array [nreps] vector [8] b;
	array [nreps] vector <lower = 0> [3] tau;
}
model {
	for (j in 1:nreps) {
	  dat[j,,2] ~ normal(a[j,1] + FE*b[j,1], tau[j,1]); // LD ~ FE
	  dat[j,,3] ~ normal(a[j,2] + FE*b[j,2] + dat[j,,2]*b[j,3], tau[j,2]); // TD ~ FE + LD
	  dat[j,,4] ~ normal(a[j,3] + FE*b[j,4], tau[j,3]); // FCT ~ FE
	  CAN ~ bernoulli_logit_glm(dat[j,,], a[j,4], b[j,5:8]); // CAN ~ FE + TD + LD + FCT
	}
}
generated quantities {
	vector [nreps] NIE_Z;
	vector [nreps] NDE_000;
	vector [nreps] NIE2_110;
	vector [nreps] NIE12_111;
	vector [nreps] TCE;
	vector [nreps] abiotic_effect;
	vector [nreps] temp_effect;
	vector [nreps] light_effect;
	vector [nreps] edge_effect;
{
	array [nreps] matrix [N, 2] LD_pred; // LD(FE = 0), LD(FE = 1)
	array [nreps] matrix [N, 3] TD_pred; // 00, 10, 11
	array [nreps] matrix [N, 2] FCT_pred; // FCT(FE = 0), FCT(FE = 1)
	array [nreps] matrix [N, 6] can_est; // FE, LD, TD(, LD()), FCT  00000, 10000 11000, 11100, 11110, 
11111

	for (j in 1:nreps) {
	  for (n in 1:N) {
		if (FE[n] == 0) {
			LD_pred[j,n, 1] = dat[j,n, 2];
			LD_pred[j,n, 2] = normal_rng(a[j,1] + b[j,1], tau[j,1]);
```

```
                        TD_pred[j,n, 1] = dat[j, n, 3];
                        TD_pred[j, n, 3] = normal_rng(a[j, 2] + b[j, 2] + LD_pred[j, n, 2]*b[j, 3], tau[j,2]);
                        FCT_pred[j,n ,1] = dat[j,n, 4];
                        FCT_pred[j,n, 2] = normal_rng(a[j,3] + b[j,4],  tau[j,3]);
                } else {
                        LD_pred[j,n, 1] = normal_rng(a[j,1], tau[j,1]);
                        LD_pred[j,n, 2] = dat[j,n, 2];
                        TD_pred[j,n, 1] = normal_rng(a[j,3] + LD_pred[j,n, 1]*b[j,3], tau[j,2]);
                        TD_pred[j,n, 3] = dat[j,n, 3];
                        FCT_pred[j,n, 1] = normal_rng(a[j,3], tau[j,3]);
                        FCT_pred[j,n, 2] = dat[j, n, 4];
                }
                TD_pred[j,n, 2] = normal_rng(a[j,2] + b[j,2] + LD_pred[j,n, 1]*b[j,3], tau[j,2]);
        }

        can_est[j,, 1] =
                inv_logit(a[j,4] + LD_pred[j,, 1]*b[j,6] + TD_pred[j,,1]*b[j,7] + FCT_pred[j,, 1]*b[j,8]);
        can_est[j,, 2] =
                inv_logit(a[j,4] + b[j,5] + LD_pred[j,, 1]*b[j,6] + TD_pred[j,, 1]*b[j,7] + FCT_pred[j,,
1]*b[j,8]);
        can_est[j,, 3] =
                inv_logit(a[j,4] + b[j,5] + LD_pred[j,, 2]*b[j,6] + TD_pred[j,, 1]*b[j,7] + FCT_pred[j,,
1]*b[j,8]);
        can_est[j,, 4] =
                inv_logit(a[j,4] + b[j,5] + LD_pred[j,, 2]*b[j,6] + TD_pred[j,, 2]*b[j,7] + FCT_pred[j,,
1]*b[j,8]);
        can_est[j,, 5] =
                inv_logit(a[j,4] + b[j,5] + LD_pred[j,, 2]*b[j,6] + TD_pred[j,, 3]*b[j,7] + FCT_pred[j,,
1]*b[j,8]);
        can_est[j,, 6] =
                inv_logit(a[j,4] + b[j,5] + LD_pred[j,, 2]*b[j,6] + TD_pred[j,, 3]*b[j,7] + FCT_pred[j,,
2]*b[j,8]);


        NDE_000[j] = mean(can_est[j,, 2] - can_est[j,, 1]);
        NIE2_110[j] = mean(can_est[j,, 4] - can_est[j,, 3]);
        NIE12_111[j] = mean(can_est[j,, 5] - can_est[j,, 4]);
        NIE_Z[j] = mean(can_est[j,, 6] - can_est[j,, 5]);
        light_effect[j] = mean(can_est[j,, 3] - can_est[j,, 2]); // same as NIE_1
        }
        temp_effect = NIE2_110 + NIE12_111;
        abiotic_effect = light_effect + temp_effect;
        TCE = NDE_000 + abiotic_effect + NIE_Z; //
        edge_effect = TCE - NIE_Z;
        }
}
```
